# Transcriptomics of *Lactobacillus paracasei*: metabolism patterns and cellular responses under high-density culture conditions

**DOI:** 10.3389/fbioe.2023.1274020

**Published:** 2023-10-12

**Authors:** Liangzhi Li, Hetian Zhang, Delong Meng, Huaqun Yin

**Affiliations:** ^1^ School of Minerals Processing and Bioengineering, Central South University, Changsha, China; ^2^ Key Laboratory of Biometallurgy of Ministry of Education, Central South University, Changsha, China

**Keywords:** *Lactobacillus paracasei*, high-density culture, transcriptomics, stress response, horizontal gene transfer

## Abstract

*Lactobacillus paracasei* has significant potential for development and application in the environmental field, particularly in addressing malodor pollution. This study aims to investigate the cellular response of *L. paracasei* B1 under high-density culture conditions. The selected strain has previously shown effective deodorizing and bacteriostatic abilities. Transcriptomics techniques are employed to dissect the nutrient metabolism pattern of *L. paracasei* B1 and its response mechanism under environmental stress. The study characterizes the functions of key differentially expressed genes during growth before and after optimizing the culture conditions. The optimization of fermentation culture conditions provides a suitable growth environment for *L. paracasei* B1, inducing an enhancement of its phosphotransferase system for sugar source uptake and maintaining high levels of glycolysis and pyruvate metabolism. Consequently, the strain is able to grow and multiply rapidly. Under acid stress conditions, glycolysis and pyruvate metabolism are inhibited, and *L. paracasei* B1 generates additional energy through aerobic respiration to meet the energy demand. The two-component system and quorum sensing play roles in the response and regulation of *L. paracasei* B1 to adverse environments. The strain mitigates oxygen stress damage through glutathione metabolism, cysteine and methionine metabolism, base excision repair, and purine and pyrimidine metabolism. Additionally, the strain enhances lysine synthesis, the alanine, aspartate, and glutamate metabolic pathways, and relies on the ABC transport system to accumulate amino acid-compatible solutes to counteract acid stress and osmotic stress during pH regulation. These findings establish a theoretical basis for the further development and application of *L. paracasei* B1 for its productive properties.

## 1 Introduction

*Lactobacillus paracasei* (also name “*Lacticaseibacillus paracasei”*), a member of the *Lactobacillus* genus, shares a close relationship with *Lactobacillus* casei and *Lactobacillus rhamnosus* in terms of phylogeny and fermentation characteristics. *L. paracasei* possesses bacteriostatic immune-regulatory properties and can metabolize and synthesize various bioproducts (Zapasnik et al., 2022). Consequently, it has significant applications in clinical medicine, food fermentation, industrial production, environmental remediation, and other fields, garnering attention both domestically and internationally (Zapasnik et al., 2022). *L. paracasei* is a prominent functional species for efficient deodorization in combating malodor pollution. *L. paracasei* secretes lipases and glycosidases and utilize nutrients from their environment to produce organic acids such as acetic acid, butyric acid, and lactic acid (Rueda-Robles et al., 2022). The resulting reduction in environmental pH inhibits the growth of other malodorous microorganisms. Additionally, the organic acids, diacetyl, and hydrogen peroxide produced by *Lactobacillus* metabolism possess broad-spectrum bactericidal effects (Rueda-Robles et al., 2022). They also produce antibacterial substances such as bacteriocins and antimicrobial peptides, effectively inhibiting the growth of pathogens like *Staphylococcus aureus, Salmonella, Legionella,* and *Escherichia coli*, as well as antagonizing the growth of most Gram-negative bacteria (Rueda-Robles et al., 2022). During fermentation, the production of diacetyl, 3-hydroxybutanone, and volatile organic acids by lactic acid bacteria results in the evaporation of fruity, aromatic flavors that physically mask odors and create a pleasant atmosphere (Verma et al., 2022). In landfills, livestock production, and solid waste composting, *L. paracasei* has been applied as a deodorizing microorganism as revealed by our previous work (Zhang et al., 2021).

Transcriptomics is the study of all transcriptomes in samples to identify and determine genes related to phenotypes by identifying genes that are differentially expressed (Liu P. et al., 2020). In the case of *Lactobacilli*, transcriptomics is used to study the functions and pathways involved in their growth and to optimize and enhance their metabolic capacity. For example, Laakso et al. investigated the changes in gene expression of *L. rhamnosus* GG when grown in industrial whey medium in a controlled bioreactor. Transcriptome and proteome analyses showed differential expression of a large number of genes and proteins, particularly those involved in carbohydrate, nucleotide, and lipid metabolism. The transition from the exponential growth phase to the stationary phase was characterized by a positive response of transporter proteins during the shift from glucose to galactose utilization. This suggests that the growth and metabolism of *L*. *rhamnosus* GG have several adaptations to nutritional conditions (Laakso et al., 2011).

In addition, the transcriptome can be used to understand the differences in the culture of key metabolic pathways of the strain in different environments and stages, and to determine the requirements of the strain for different nutrients (Liang et al., 2020). Feng’s study on the transcriptional differences during the growth of *Lactobacillus paracasei* PC-01 showed that the transcript levels of amino acid metabolism pathways related to aspartic acid and glutamic acid gradually increased after the logarithmic growth period, and therefore these nutrient substrates could be supplemented with the fermentation process in order to strengthen the growth ability of the strain (Feng, 2021). Similarly, Jiao investigated the sugar metabolism pathway of *Enterococcus faecalis* under aerobic respiration and found that the genes coding for the aerobic respiration chain were constitutively expressed. These genes mainly drove the conversion of lactate into ethanol by regulating key genes of the pyruvate and butyric acid metabolism pathway. By optimizing the culture medium through the addition of hemoglobin, the final number of viable bacteria of LD33 of *E. faecalis* increased significantly (Jiao, 2016). Thus, transcriptome can provide valuable insights and guidance for the industrial production of Lactobacilli by resolving their nutritional consumption pattern, optimizing the growth medium, reducing non-essential nutritional costs, and increasing the amount of growth or products produced.

Furthermore, Lactobacilli often encounter environmental stress during the growth process, such as acid, salt, heat, and oxygen stress (Cataldo et al., 2021). Under these stresses, lactic acid bacteria initiate stress responses to mitigate the damages. For example, Lee et al. found that *Lactobacillus reuteri* overexpressed genes related to glucose metabolism under low pH regulation through transcriptome analysis. This suggests that higher energy metabolism is required under low pH conditions, and a large number of intermediary metabolites for energy metabolism will be produced to maintain additional energy supply (Lee et al., 2010). Similarly, *Lactobacillus fermentum* IMDO can alter major metabolic pathways, such as converting fructose to mannitol and raising acetate, to ensure energy supply under acid stress (Vrancken et al., 2008). Under salt stress, *Lactobacillus plantarum* FS5-5 decreases the expression levels of genes related to carbohydrate metabolism, amino acid transport and metabolism, vitamin synthesis, and nucleotide metabolism. This behavior may be a protective measure to limit metabolic acquisition under stressful conditions (Song et al., 2017). By studying the stress responses of Lactobacilli and implementing corresponding protective measures, the survival rate of Lactobacilli in industrial production can be improved.

*Lactobacillus paracasei* exhibits variations in substance and energy metabolic activities during different stages of fermentation and under different culture conditions (Zhang et al., 2021). These variations are driven by complex metabolic networks that involve various biochemical processes, including cellular metabolism and genetic information processing. One of the main challenges in the industrial application of *Lactobacillus* is the limited theoretical understanding of cellular metabolism and regulation. The mechanisms of stress response during growth and metabolism are particularly understudied. Advances in transcriptomics technology can help characterize the growth and nutrient metabolic patterns in regulatory lactic acid bacteria, shedding light on the stress mechanisms operating under environmental stress. Moreover, these insights can contribute to a comprehensive understanding of the physiological properties of lactic acid bacteria, ultimately improving their production performance in fermentation processes.

By using transcriptomics to analyze the metabolic network of *L. paracasei*, we can identify differences in gene expression between samples at different fermentation stages or culture conditions. This analysis helps us understand the dynamic fluctuations in material and energy metabolism, as well as the diversity of cellular metabolism and functions. In this study, we cultured *L. paracasei* B1, a strain isolated in our previous study (Zhang et al., 2021), under two different fermentation modes, under or without fermentation optimization. We considered the mid-log phase and stationary phase as crucial time points for investigation. We conducted transcriptome sequencing on samples at different growth stages within the same fermentation mode, as well as between different fermentation modes at the same growth stage. Our aim was to analyze the correlation of gene expression patterns across samples during fermentation processes and identify differentially expressed genes (DEGs).

## 2 Materials and methods

### 2.1 Sampling during fermentation

This study utilized two distinct fermentation modes of *Lactobacillus paracasei* B1, a strain isolated in our previous study (Zhang et al., 2021), over a period of 40 days: one before fermentation optimization and one after fermentation optimization. Experiment 1 utilized a simple batch culture, while Experiment 2 implemented the culture conditions after fermentation optimization as outlined in our previous study (Zhang et al., 2022). The specific control parameters that differed between the two fermentation modes are summarized in Table 1. The mid-log phase (μ = μ _max_) and the stationary phase (μ = 0) were chosen as the critical time points for sampling during the fermentation process. For Experiment 1, the sampling points were designated as CK1 and CK2, while for Experiment 2, they were labeled as T1 and T2. The samples at each point were collected in triplicate.

**TABLE 1:** Difference of experimental fermentation control parameters.

Cultivation conditions	Experiment 1	Experiment 2
Temperature	35°C	The first 8 h were kept at 40°C and then the temperature was changed to 35°C
pH	—	5.5 ± 0.25
Supplementation	—	Feedback flow supplementation (sucrose + growth factors)

Note: “-“ means no control.

Growth curves and specific growth rate curves were generated by monitoring OD_600_. Samples were collected during the logarithmic growth phase (μ = μ _max_) and the stationary phase (μ = 0), with three replicates for each experiment.

Both sets of fermentation experiments were conducted in a 50 L fermenter using the optimized combination of fermentation medium: sucrose 37.8 g/L, yeast extract 33.7 g/L, K_2_HPO_4_ 7 g/L, KH_2_PO_4_ 7 g/L, MgSO_4_ 0.2 g/L, MnSO_4_ 0.02 g/L, Tween-80 0.1 g/L, glycerol 0.1 g/L, Vitamin B_3_ 0.01 g/L, and cysteine hydrochloride 0.2 g/L. Other fermentation parameters, including temperature, pH, bacterial density, and residual sugar, were also measured.

### 2.2 Sample preparation

The fermentation broth was examined microscopically, and the bacterial count in each sample was required to be ≥1×10^8^. A 1.5 mL aliquot of the fermentation broth was transferred to a 2 mL RNase-free centrifuge tube with a screw cap and a pointed bottom. The tube was then centrifuged at 14,000 g for 10 min at 4°C. After centrifugation, the supernatant was discarded, and the bacterial pellet was rapidly frozen in liquid nitrogen for at least 1 h before being stored at −80°C.

### 2.3 Sequencing library construction and high-throughput sequencing

Total RNA was extracted from the samples following the instructions provided with the TRlzol reagent. Genomic DNA was removed using DNaseI. The quality of RNA was assessed using a 2100 Bioanalyzer, and its concentration and purity were determined using a NanoDrop 2000. High-quality RNA samples were then utilized for sequencing library construction.

RNA-specific libraries were reconstructed using Illumina TruSeq RNA Sample Preparation Kit, employing 5 μg of total RNA per set. The RiboZero rRNA Removal Kit was used to eliminate rRNA, and mRNA was fragmented using Fragmentation buffer. Subsequently, cDNA reverse transcription synthesis, end-pairing, A-base separation, and junction ligation were performed based on the Illumina manual. cDNA fragments between 200 and 300 bp were selected through electrophoresis on a 2% agarose gel. These fragments were then amplified using Taq high-fidelity polymerase (Q5 Hot Start High-Fidelity DNA Polymerase, NEB) for 15 PCR cycles. Prior to sequencing, the quality of the libraries was assessed using a NanoDrop microspectrophotometer and Labchip bioanalyzer. Finally, the paired libraries (150 bp × 2) were sequenced using an Illumina NovaSeq 6000 sequencer (LC-Bio Technology Co., Ltd., Hang Zhou, Zhejiang Province, China).

The high-quality genome of *L. paracasei* B1 was used as a reference for mapping RNA reads. The genomic DNA of *L. paracasei* B1 was isolated using the protocol from Platero et al. (Martín-Platero et al., 2007) and sequenced on PacBio Sequel II platform using a single SMRT cell at the LC-Bio Technology Co., Ltd., Hang Zhou, Zhejiang Province, China. The genome was assembled using Flye v2.7 (Kolmogorov et al., 2020). The ORFs were predicted using Prodigal (Hyatt et al., 2010).

The transcriptome raw data have been make available in the NCBI Sequence Read Archive (SRA) database at https://www.ncbi.nlm.nih.gov/sra/PRJNA1020974. The genome assembly of *Lactobacillus paracasei* is available at https://ngdc.cncb.ac.cn/bioproject/browse/PRJCA020011.

### 2.4 Bioinformatics analysis

#### 2.4.1 Raw data statistics and quality control

Raw data obtained from Illumina high-throughput sequencing downstream often contain junction sequences, low-quality sequences, too short sequences, and sequences containing too many N bases, which can impact the accuracy of subsequent assembly and comparison. Therefore, it is necessary to cut and filter the data to obtain high-quality clean data. The software used for this purpose is fastp (v0.23.2, https://github.com/OpenGene/fastp). The fastp filtering QC requirements include discarding sequences with less than 50 bases and limiting the number of N bases to 6. Additionally, the quality value should be greater than or equal to Q15 to be considered qualified. After filtering through the fastp quality control sequence, the data was deemed of high quality (Chen et al., 2018).

#### 2.4.2 Reference sequence comparison

To assess the quality of the cleandata, it was compared with the genome sequence of *L. paracasei* B1. The genome sequence was derived from the whole genome data of the strain that was sequenced and spliced in our laboratory. The comparison was performed using STAR software (v2.7.9a, https://github.com/alexdobin/STAR/) (Dobin et al., 2013).

#### 2.4.3 Gene expression quantification and analysis

The quantification of gene expression levels was carried out using RSEM software (v1.3.3, http://deweylab.biostat.wisc.edu/rsem/). This approach helps to remove the effects of RNA sequencing depth and gene length (Li and Dewey, 2011).

Transcript expression was quantified after importing the bam files generated during the bowtie comparison process into RSEM. The gene expression was then calculated. RSEM provides quantitative results in units of FPKM, read counts, and TPM. For this study, FPKM was used to examine the quantitative level of gene expression.

#### 2.4.4 Differential expression gene screening

Differential expression analysis enables the comparison of gene changes across different experimental groups. In this study, we conducted comparisons of transcriptome expression levels within and between experimental groups. Specifically, we compared transcriptome expression levels during the mid-log phase and stable period, while keeping the fermentation mode treatment constant. These specific comparisons were CK1 vs. CK2 in Experiment 1 and T1 vs. T2 in Experiment 2. Furthermore, we also compared transcriptome expression levels between the log phase and stable period, considering two different fermentation modes: CK1 vs. T1 and CK2 vs. T2.

#### 2.4.5 Functional enrichment analysis of differentially expressed genes

There were three biological replicates for each treatment in this experiment. Differential gene expression analysis was performed using DESeq2 software (v1.36.0, https://bioconductor.org/packages/release/bioc/html/DESeq2.html). DESeq2 employs a statistical model to predict the significance of gene differences. The screening thresholds used were |Log2Fc| ≥ 1 and *p*-value <0.05 (Li and Dewey, 2011).

In this study, we performed sequence functional annotation and classification using EggNOG mapper v2 (http://eggnog-mapper.embl.de/). This tool utilizes the HMMER algorithm for sequence matching. After annotating the sequences with EggNOG-related classification systems such as COG, KEGG, and GO (Cantalapiedra et al., 2021), we conducted enrichment analysis.

Furthermore, we compared the differentially expressed genes with homologous genes in the KEGG Orthology database (https://www.kegg.jp/kegg/ko.html) to determine the localization and function of the differentially expressed genes in the metabolic pathway. This analysis allowed us to understand the response of *L. paracasei* B1 metabolism regulation mechanism to environmental changes during the fermentation process.

The horizontal gene transfer (HGT) profile, “mobileome”, of Lactobacillaceae was identified as follows: The Integrated Microbial Genomes Annotation Pipeline (IMGAP) v.5.0 (Markowitz et al., 2010) under default mode was used to identify horizontally transferred genes (HTGs) in the genomic sequences of Lactobacillaceae available at Genbank/IMG database, as conducted in previous studies (Li et al., 2023). It used the following criteria to determine which genes inside the trialed genomes were horizontally transferred from remote descendants: genes with the finest BLASTP matches (most significant bit scores) or over 90% of the best hits discovered beyond the phylogenetic clade of the trialed genome (i.e., from remote phylum, class, etc.) and with lower-scoring matches or no hits within the original phylogenetic clade of the trialed genome. BLASTN was then applied to map the DEGs *L. paracasei* B1 onto the “mobileome” of Lactobacillaceae to detect DEGs putatively affected by the HGT process with threshold: sequence identity >90%, E-value < 1e^−10^.

## 3 Results and discussion

### 3.1 Experimental sampling points and grouping

The effects of the two fermentation modes on the growth of *L. paracasei* B1 varied significantly. Experiment 2, which was optimized for fermentation control, had a maximum growth density of 2.221, while Experiment 1 had a maximum growth density of 1.295.

**FIGURE 1:**
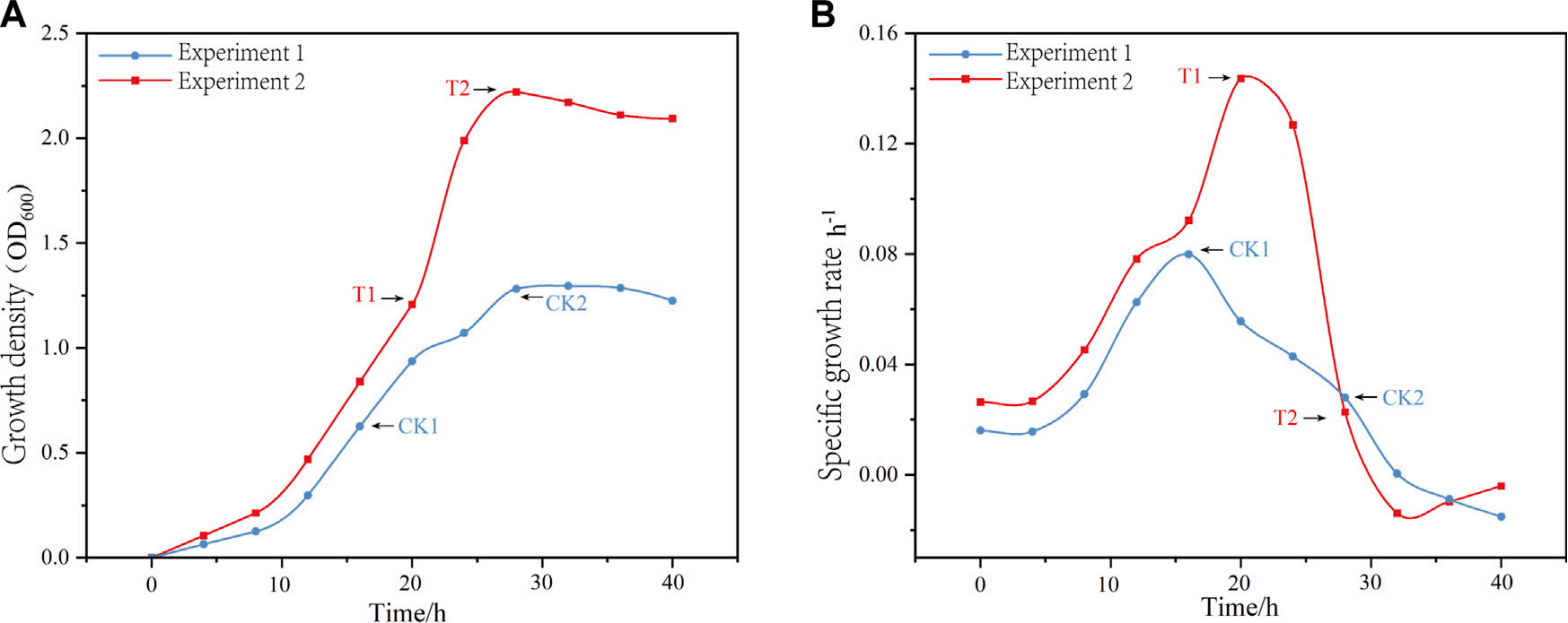
The variations in growth density **(A)** and growth rate **(B)** over time, along with the corresponding sampling points for two fermentation modes, labeled as CK1, CK2, T1, and T2.

The key time points selected for the fermentation process were the mid-log phase (μ = μ _max_) and the stationary phase (μ = 0). In Experiment 1, the mid-log phase occurred at 16 h and the stationary phase occurred at 28 h. These time points were labeled as CK1 and CK2, respectively. In Experiment 2, the mid-log phase occurred at 20 h and the stationary phase occurred at 28 h. These time points were also labeled as T1 and T2, respectively. Figure 1 shows the changes in the growth density of the two fermentation modes and the moments of the sampling points. Among the four sampling points, the pH of CK2 was the lowest at 3.42, followed by CK1 at 4.53, and T1 and T2 both at 5.5.

### 3.2 Assessment of total RNA purity and integrity

RNA is susceptible to degradation during sample collection, processing, and isolation, resulting in a decline in its integrity and quality. Moreover, low-quality RNA can significantly affect the accuracy of gene expression levels and differential analysis (Romero et al., 2014). Hence, it is crucial to implement rigorous quality control measures to evaluate the quality of RNA extracted from the samples, including assessing its purity and integrity. The outcomes of these assessments are presented in Supplementary Table S1 and Supplementary Figure S1.

### 3.3 Sequencing and filtering results

The raw data from each sample underwent fastp filtering to obtain an average of more than 4 million clean reads. The sequencing evaluation report for the sample clean reads is shown in Supplementary Table S2. Q20 and Q30 are the proportions of total bases in the samples with quality scores ≥20 and ≥30, respectively. The Q20 and Q30 across all samples reached more than 97% and 92%, respectively. The GC content represented the GC content, which was more than 46% on average. These results indicate that the clean reads after quality control (QC) were in compliance with the requirements.

### 3.4 Whole genome sequence mapping

The filtered data from all samples were mapped to the assembled whole genome sequence of the strain. The Supplementary Table S3 shows the number of uniquely mapped reads, which indicates the total number of sequences that were successfully aligned to a unique position on the genome. On average, the unique mapping rate was 94.64%. The unmapped reads and the unmapped reads ratio represent the total number and percentage of sequences that could not be aligned to the genome, with an average of 5.37%. These results suggest that the sequenced sequences have a high alignment rate with the genome sequences, which makes them suitable for subsequent analysis.

### 3.5 Quantification of gene expression

The quantification of gene expression involves considering the number of reads that are present in a gene region. However, it is important to note that this number is influenced by factors such as gene length and sequencing depth. Additionally, the read data exhibit a positive correlation with both gene length and sequencing depth. Given that these factors can vary across each sample, it is necessary to normalize them. The Fragments Per Kilobase of exon model per Million mapped fragments (FPKM) is used for this purpose. FPKM is calculated by dividing the number of normalized reads by the length of the gene. A higher FPKM value indicates a higher level of gene expression (Zhao et al., 2021). In Supplementary Table S4, the FPKM values are divided into five intervals, and the number of genes falling within each expression level interval is counted. This provides statistics on the distribution of genes across different levels of expression. The distribution of gene expression density is commonly employed in illustrating the distribution of expression levels in individual samples. As depicted in Supplementary Figure S2, the *x*-axis of the distribution plot represents log_10_(FPKM+1), while the *y*-axis denotes the density of genes whose expression falls within a given interval. The 12 samples exhibit a relatively consistent distribution of expression levels, with more concentrated peaks. This suggests that the expression of each sample is largely similar.

### 3.6 Sample clustering and correlation analysis

**FIGURE 2:**
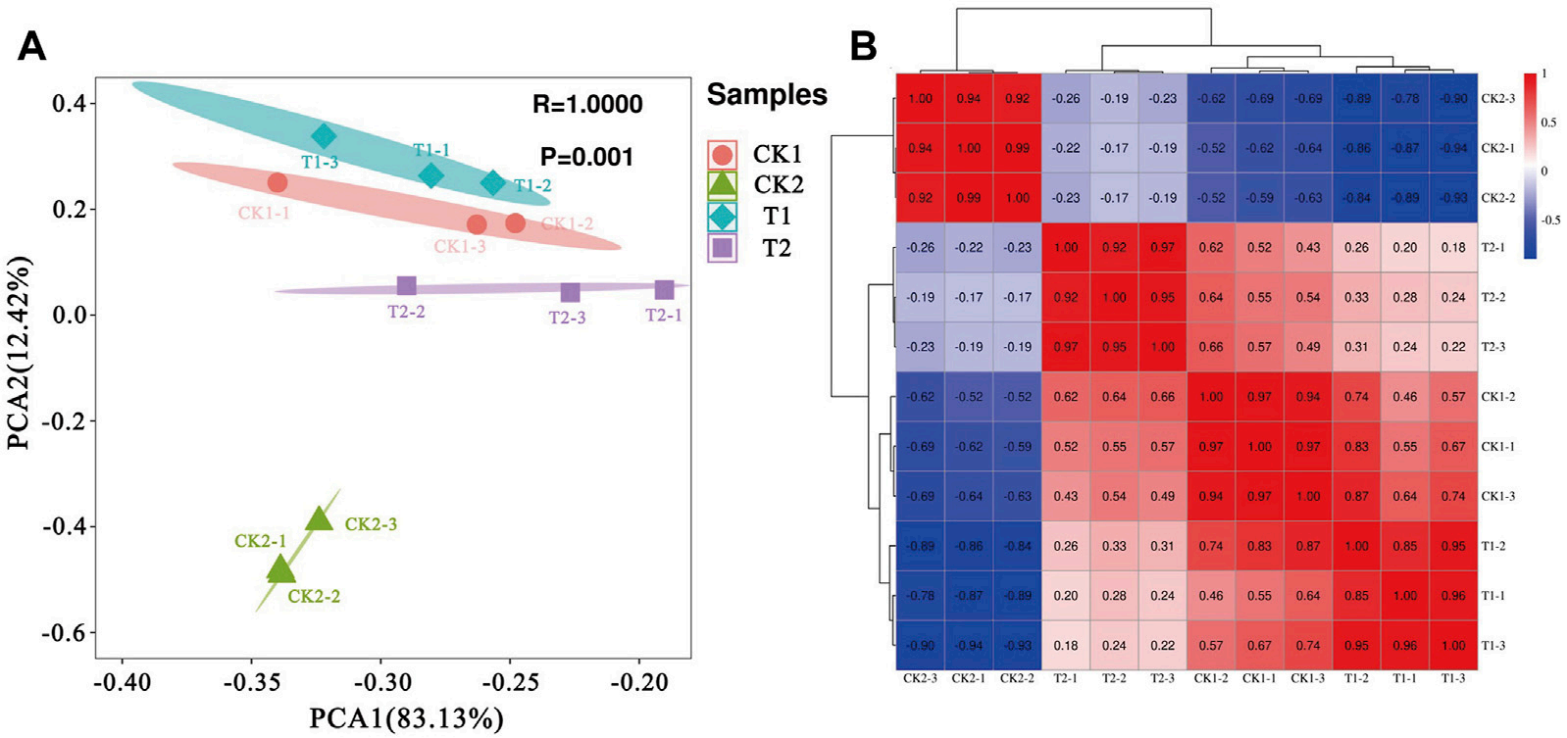
Sample principal component **(A)** and correlation analysis **(B)**.

Principal component (PCA) and correlation analyses were conducted on the samples based on gene expression. The PC analysis in Figure 2A shows that PC1, representing the first principal component, contributes 83.13% to the overall variance, while PC2, representing the second principal component, contributes 12.42% to the overall variance. Together, PC1 and PC2 account for 95.55% of the overall variance. The samples from the four sampling points are observed to be clustered based on PC1 and PC2, with no outlier samples. This indicates good sample repeatability. Additionally, there is no overlap when setting 95% confidence intervals for each sampling point, suggesting significant differences between the samples. Among them, CK2 is the furthest away from CK1, T1, and T2, indicating a large variability or variance in CK2 compared to the other samples. This suggests that CK2 has distinct transcriptome characteristics and different gene expression patterns.

The correlation analysis in Figure 2B confirms the PCA results. The samples from the same growth stage sampling point show obvious clustering, with CK2 having the lowest correlation with the other sampling points. When comparing two sampling points at a time, samples from CK1 and T1, both sampled at the mid-log phase, show higher correlation. On the other hand, samples from the mid-log phase and stabilization time under the same fermentation mode show lower correlation.

The results suggest that there were significant differences in gene expression patterns at each stage of fermentation before and after optimization of *L. paracasei* B1. There is a high correlation between samples in the middle of the logarithmic phase in both fermentation modes, indicating similar gene expression patterns. The effect of fermentation optimization is smaller during the middle and early stages of fermentation, as evidenced by the low correlation and significant difference in gene expression patterns in the stationary phase of both fermentation modes. This may be due to the fact that in the late stage of fermentation without optimization, the strains experience increased environmental stresses, leading to significant changes in gene expression to enhance environmental adaptability and stress resistance. A similar pattern is observed between the mid-log phase and stable period under unoptimized fermentation, where increasing environmental stress leads to low correlation and significant differences in gene expression. On the other hand, after fermentation control optimization, there is a higher correlation in gene expression patterns between the mid-log phase and stable period, which may be attributed to the reduced environmental fluctuation through culture addition optimization and the smooth transition of gene expression patterns between different fermentation stages.

### 3.7 Screening of differentially expressed genes

**FIGURE 3:**
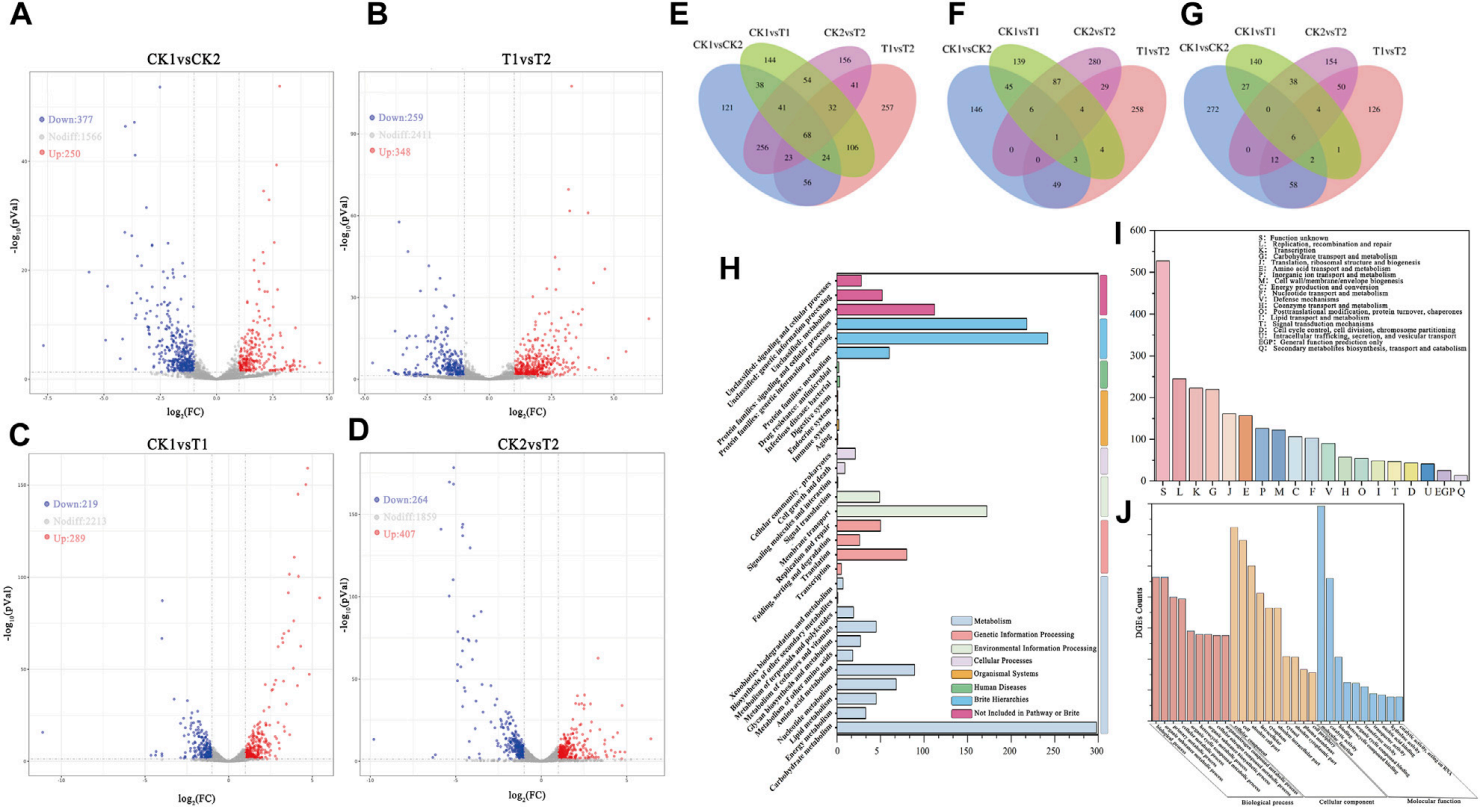
Volcano map of differentially expressed gene between samples: **(A)** CK1 and CK2, **(B)** T1 and T2, **(C)** CK1 and T1, **(D)** CK2 and T2. **(E)** Differentially expressed genes Venn diagram between samples. Venn diagrams of upregulated DEGs **(F)** and Downregulated DEGs **(G)** between samples. **(H)** KEGG classification histogram of DEGs. **(I)** DEGs GO classification histogram (TOP20). **(J)** DEGs GO classification histogram (TOP10 for each category).

Genes with significant changes were identified as DEGs through differential gene analysis by comparing gene expression between the two groups. The number of significantly upregulated and downregulated genes in the samples was determined using DESeq2 to analyze the expression matrix of the count values (Figures 3A–D).

Most expressed genes did not exhibit significant changes between samples (|Log_2_Fc| < 1). A total of 627 DEGs were detected in CK1 vs. CK2, including 377 upregulated genes and 250 downregulated genes. Similarly, a total of 607 DEGs were detected in T1 vs. T2, including 259 upregulated genes and 348 downregulated genes. Although the number of differentially expressed genes was similar in both fermentation modes, the trends of upregulation and downregulation were opposite. This may indicate opposite characteristics in gene expression trends between the two groups of experiments before and after the optimization of CK2 and T1 vs. T2 culture conditions. In CK1 vs. T1, a total of 507 DEGs were detected, with 289 genes upregulated and 219 genes downregulated. In CK2 vs. T2, a total of 671 DEGs were detected, with 407 genes upregulated and 264 genes downregulated. This suggests that more gene expression was significantly induced in the fermentation mode under unoptimized culture conditions as environmental stresses increased over time.

After tallying the number of DEGs at each sampling point, the DEGs that were common and distinct among each sample were further analyzed to identify common and specific DEGs in the two fermentation modes. This analysis was illustrated in Figure 3E using Venn diagrams. Among all samples, a total of 68 shared DEGs were found among the comparison group. Moreover, there were 171 DEGs that were shared in both CK1 vs. CK2 and T1 vs. T2 fermentation modes. It is shown that these 68 shared DEGs exhibit a high correlation in their response to the changing environment. Even slight changes in the culture environment induced alterations in the expression levels of these genes. After removing these 68 shared DEGs, all the remaining DEGs may be associated with the phase transition during fermentation. The remaining set of 103 DEGs, after removing the 68 shared DEGs, could be potentially linked to the fermentation phase transition. Furthermore, in CK1 vs. CK2, there were 121 specific DEGs, while in T1 vs. T2, there were 257 specific DEGs. These findings suggest that these two different sets of DEGs are highly specific and exhibit varying gene expression patterns due to environmental stress or fermentation optimization.

There were 208 shared DEGs between the comparison groups CK1 vs. T1 and CK2 vs. T2. Additionally, there were 312 DEGs specific to CK1 vs. T1 and 476 DEGs specific to CK2 vs. T2. These DEGs represent the differential gene expression caused by fermentation optimization before and after the two growth phases. The shared DEGs play a global role in the fermentation process, while the specific DEGs in CK1 vs. T1 and CK2 vs. T2 represent differentially expressed genes influenced by stress and fermentation optimization in the mid-log phase and stationary phase of fermentation, respectively. The specific DEGs among different samples are considered key genes and analyzing them can provide insights into the response of *L. paracasei* B1 to environmental stresses during fermentation and the gene regulatory pathways influenced by fermentation optimization. This analysis can be valuable for optimizing future fermentation processes. Figures 3F, G illustrate the overlap of upregulated and downregulated genes among the DEGs in the different comparison groups. Specifically, CK1 vs. CK2 had 53 upregulated DEGs and 78 downregulated DEGs, while T1 vs. T2 had 78 upregulated DEGs and 78 downregulated DEGs. These changes in gene expression are hypothesized to reflect alterations in metabolic pathways during different growth stages. In CK1 vs. CK2, there were 146 specifically upregulated DEGs and 272 specifically downregulated DEGs. In contrast, T1 vs. T2 had 258 specifically upregulated DEGs and 126 specifically downregulated DEGs. Conversely, the optimization of fermentation during T1 vs. T2 resulted in most genes being expressed during the growth period to facilitate growth and metabolic processes for faster growth and proliferation. Further analysis is needed to determine if these DEGs are associated with specific biological pathways or functional modules in order to provide a comprehensive explanation of these results. Further examination of DEGs in CK1 vs. T1 and CK2 vs. T2 revealed that there were 98 shared upregulated DEGs and 48 shared downregulated DEGs. Furthermore, there were 197 specific upregulated DEGs and 299 specific downregulated DEGs in CK1 vs. T1, as well as 295 specific upregulated DEGs and 181 specific downregulated DEGs in CK2 vs. T2. These findings indicate that *L. paracasei* B1 exhibits varying gene expression patterns and engages in different metabolic pathways at different growth stages within the same fermentation mode and among different fermentation modes at the same growth stage. These variations reflect changes in the expression levels of functional genes, enabling microorganisms to meet their own growth, development, and adaptation needs in response to the changing environment during growth stage transitions and in the presence of different culture conditions.

The differentially expressed genes identified through screening were categorized based on genome annotation information or compared to database annotation information. Subsequently, key pathways and molecular mechanisms involved in the biometabolic process were examined to gain a better understanding of the growth and metabolic mechanisms of *L. paracasei* B1, as well as its stress response mechanisms under environmental stress. This analysis provides a theoretical foundation for optimizing the metabolic capacity of *Lactobacillus* and enhancing its environmental tolerance.

### 3.8 Annotation of differentially expressed genes

The KEGG database (Kyoto Encyclopedia of Genes and Genomes) is a comprehensive database that integrates functional information related to the genome, gene products, and metabolic pathways (Kanehisa and Sato, 2020). It provides systematic analysis of the metabolic pathways of gene products and compounds within the cell, as well as the functions of these genes. The results of the comparison with the KEGG database are presented in Figure 3H. At the class I level, the differentially expressed genes were primarily classified into Metabolism (34.74%), Brite Hierarchies (27.79%), Environmental Information Processing (11.86%), and Genetic Information Processing (8.61%). At the class II level, the differentially expressed genes were mainly categorized into Carbohydrate Metabolism (15.93%), Genetic Information Processing (12.63%), Membrane Transport (9.19%), Amino Acid Metabolism (4.75%), and Translation (4.27%). According to the KEGG annotation results, the observed differences in gene expression across different fermentation modes and growth stages were primarily associated with carbohydrate metabolism, protein synthesis, membrane transport, and gene expression regulation.

The COG (Clusters of Orthologous Groups) database is a functional annotation database that categorizes homologous proteins based on their function and evolutionary relationships (Galperin et al., 2021). By comparing differentially expressed gene sequences with those in the COG database, one can infer the biological function of the encoded protein. The results presented in Figure 3I indicate that a majority of the sequences were annotated, with only a small percentage being functionally unknown (21.9%). The annotated sequences were associated with various functions, such as replicative recombination and repair (10.2%), transcription (9.26%), carbohydrate transport and metabolism (9.10%), amino acid transport and metabolism (6.52%), and inorganic ion transport and metabolism (5.23%). These results suggest that the differences in growth and evolutionary relationships among the sampling sites are not fully understood. It can be inferred that the physicochemical variations in the growth environment between the sampling sites lead to changes in the nutritional requirements of the cells. Additionally, there is evidence of DNA damage, mutation and repair during the fermentation process, as indicated by the prevalence of the “replication, recombination, and repair” category.

Figure 3J depicts the results of the comparison of differentially expressed genes with the Gene Ontology (GO) database, which serves as a standardized functional annotation tool for genes, proteins, and other biomolecules (Blake et al., 2015). The DEGs in this study were annotated to 1,580 GO Terms, which were categorized into Biological Process (BP, 61.4%), Molecular Function (MF, 19.69%), and Cellular Component (CC, 18.9%). The transcriptome of the strain as a whole was broadly regulated and influenced by the two fermentation modes and the shift in the growth phase of the fermentation process.

### 3.9 Enrichment analysis of DEGs between CK1 and CK2 groups

**FIGURE 4:**
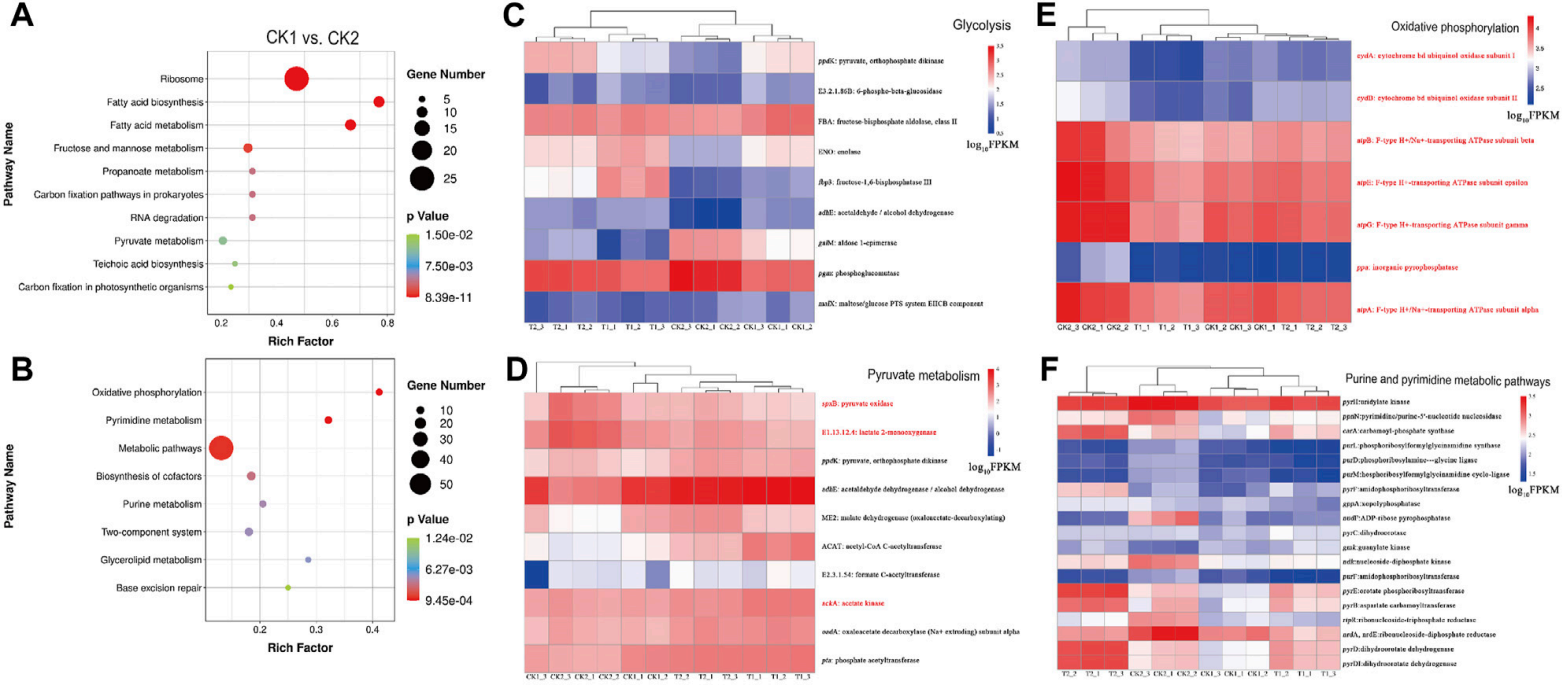
Enrichment bubble diagram of DEGs between CK1 vs. CK2: **(A)** upregulation, **(B)** downregulation. Abundance of DEGs in pathways: **(C)** Glycolysis, **(D)** Pyruvate metabolism, **(E)** Oxidative phosphorylation and **(F)** Purine and pyrimidine metabolic pathways. Genes associated with aerobic respiration and upregulated in *L. paracasei* B1 under low pH conditions are marked with red color.

The comparison of CK1 vs. CK2 groups represents the differentially expressed genes that exhibited upregulation or downregulation during the mid-log phase and stable period in the experimental group prior to the optimization of the fermentation culture conditions. Figure 4A illustrates the upregulation of genes associated with ribosomes, fatty acid synthesis and metabolism, fructose and mannose metabolism, propionic acid metabolism, pyruvic acid metabolism, phospholambanic acid synthesis, and the carbon fixation pathway during the mid-log phase.

Ribosomes are organelles responsible for protein synthesis. Among the ribosomal proteins, EF-Tu assists in ribosome and tRNA recognition and pairing, regulating the rate and precision of protein synthesis through the formation of the intermediate isopeptidyl-tRNA-EF-Tu complex. Additionally, the auxiliary secretory channel protein SecY is responsible for the transmembrane translocation of precursor proteins (Gumbart et al., 2009; Liu et al., 2014). In the middle of the logarithmic phase, the genes encoding these two proteins (RP-S10, RP-L3, RP-S17, RP-L14, etc.) were significantly upregulated, as shown in Supplementary Figure S3. This suggests that *L. paracasei* B1 rapidly produces a large number of ribosomes to meet the demands of both rapid proliferation and metabolic load during the logarithmic phase. However, as the cell enters the stationary phase, proliferation gradually ceases and metabolic activities slow down substantially. Consequently, the demand for ribosomes decreases and, accordingly, the expression levels of ribosome-related genes are reduced. The T1 vs. T2 comparison group yielded similar results, with the expression levels of ribosome-related genes decreasing from high to low from the middle of the logarithmic phase to the stationary phase. This trend aligns with the bacteria’s proliferation and growth attenuation process, suggesting that the expression levels of ribosomal genes can serve as indicators of the growth process (Liu T. J. et al., 2020).

Most carbohydrate metabolism pathways, such as fructose and mannose metabolism, propionate metabolism, pyruvate metabolism, and glycolysis, are significantly enriched and expressed at high levels during the logarithmic growth phase. These metabolic pathways produce ATP, NADH, and various metabolites. Additionally, glycolysis, pyruvate metabolism, and sugar source metabolism are closely linked, providing a large amount of energy and metabolites for further metabolism of other products. The level of pyruvate metabolism is often positively correlated with the efficiency of sugar source utilization, and is an important parameter for growth (Milanovic et al., 2012). Therefore, the abundance of DEGs in the carbon metabolic pathway reflects the efficient utilization of sugar sources and active metabolic activities of *L. paracasei* B1 during its rapid growth and reproduction (Zhan et al., 2023). Previous studies have shown that the transfer of such carbon metabolic genes has led to the domestication of *Penicillium* species. This transfer enhances their capacity to use monosaccharides and optimize carbon absorption for fermentation, allowing them to survive in environments with nutrient deficiencies (Ropars et al., 2020).

On the other hand, the downregulation of genes associated with glycolysis and pyruvate metabolism in the post-stationary phase (Figure 4B), when compared to the mid-log growth phase, can be attributed to two main factors. Firstly, the growth rate of the strain weakens and the intracellular demand for substances and energy decreases. Secondly, when the culture environment has a lower pH, it leads to the formation of a proton gradient difference between the intracellular and extracellular environments, resulting in an increased intra-proton flux. This disrupts the stable internal environment required for glycolysis and pyruvate metabolism (Bedoya-Correa et al., 2019). In this study, we hypothesize that the downregulation of gene expression in these pathways is mainly affected by the latter factor, as a significant amount of energy is needed to maintain intracellular stability and to transport protons to the extracellular environment using H^+^-ATPase in an acidic environment (Zhang et al., 2016).

Lipophosphatidic acid is an important component of the cell membrane of Gram-positive bacteria. Upregulation of lipophosphatidic acid synthesis pathway genes in the middle of the logarithmic growth phase increases the content of lipophosphatidic acid in the cell wall, enhancing cell wall density and reducing proton permeability. This prevents the inward flow of H^+^ and improves acid tolerance (Wei et al., 2021). The synthesis of lipophosphatidic acid is correlated with the expression of genes involved in carbohydrate metabolism pathways, but their expression is downregulated in the stable growth phase. Lipophosphatidic acid (LPA) synthesis is reduced despite the lower environmental pH during the steady state phase. Synthesis of LPA necessitates significant uptake of raw materials and energy, including diacylglycerol (DAG), uracil diphosphoglucose (UDP-Glc), and uracil diphosphogalactose (UDP-Gal), as illustrated in Supplementary Figure S4. However, in the later stages of fermentation, bacteria utilize primary metabolites more conservatively, which requires the cell to enhance other antacid mechanisms to maintain intracellular homeostasis during growth and metabolism (Wiegand et al., 2013).

After entering the stationary phase, the majority of differentially expressed genes showed enrichment in various pathways, including oxidative phosphorylation, pyrimidine and purine metabolism, two-component system, glycerophospholipid metabolism, and base excision repair. These genes were significantly upregulated (Supplementary Figure S5).

The two-component system (TCS) is a crucial mechanism of cell signaling and metabolic regulation that is widely present in bacteria. It senses external environmental changes and stimuli through membrane receptor kinases and transmits intracellular response regulator molecules to regulate the physiological activities of cells. This enhances their adaptability to the environment (Tiwari et al., 2017). The enrichment of genes related to the two-component system during the stationary phase reflects the positive response of *L. paracasei* B1 to environmental changes (Huynh and Stewart, 2011). Gene enrichment in phosphorus limitation, the acidic environment, redox signaling, and CAMP signaling and response pathways (as presented in Supplementary Figure S5), suggests that the strain is capable of sensing phosphorus shortage, changes in pH, and redox potential through two-component systems (TCS) during the stationary phase. This activation initiates a cascade of responsive reactions that aid microorganisms in adapting to environmental changes, including phosphorus allocation, resistance to acid-induced stress, and aerobic respiration.

Cationic antimicrobial peptide (CAMP) is a class of small molecular weight peptides with a positive charge. In addition to triggering cellular signaling processes, such as the activation of transcription factors, alteration of gene expression, and regulation of cellular metabolism, CAMP also helps to enhance microbial adaptation to the environment and improve their ability to survive and resist external stresses (Yang et al., 2013; Zhao et al., 2022). The rejection behavior of CAMP suggests the presence of a high concentration of CAMP accumulation, which can trigger microbial adaptive responses, such as over-expression or damage. The DltAC gene is involved in encoding the D-alanine-polyphosphatidylcholine ligase subunit protein. It catalyzes the formation of positively charged D-alanine-phosphatidylcholine, which repels CAMP and helps to maintain stable endogenous levels of CAMP (Li et al., 2007).

In the later stages of fermentation, when there is an accumulation of organic acids resulting in an acidic environment, certain strains of bacteria, such as *Lactobacilli*, undergo metabolic reconfiguration to adapt to these conditions. This adaptation is necessary because low pH inhibits glycolysis and pyruvate metabolism, requiring alternative pathways for energy production (Koponen et al., 2012).

Most *Lactobacilli* typically respire anaerobically, but some strains possess genes encoding the respiratory electron transport chain, including *cydAB* manipulators that can produce heme-dependent cytochromes. This enables aerobic respiration in the presence of oxygen, heme, and menaquinone (Duwat et al., 2001). Aerobic respiration in *Lactobacilli* leads to increased expression and activity of enzymes such as NADH oxidase and NADH peroxidase, enhancing their ability to compete with lactate dehydrogenase for NADH molecules. This redirection of pyruvate metabolism towards the production of acetate, ethanol, and diacetyl instead of lactate improves their adaptability (Pedersen et al., 2008).

Enrichment analysis of CK1 and CK2 revealed that *L. paracasei* B1 was enriched for several DEGs in the oxidative phosphorylation pathway, which is crucial for energy metabolism. These DEGs included the *atpCDG* gene, responsible for encoding F-type ATP synthase, and the *cydAB* gene, responsible for encoding cytochrome *bd* ubiquinone oxidase. From this, it can be inferred that *L. paracasei* B1 has the ability to obtain energy through a combination of aerobic and anaerobic respiration (Papadimitriou et al., 2016). It has been shown that *Acetobacter* regulates intracellular sodium ion content through the horizontally acquired ATP synthase, which is associated with stress resistance (Matsutani et al., 2020). Additionally, the genes involved in the pyruvate metabolic pathway showed significant upregulation during the steady state phase. For instance, the gene encoding pyruvate oxidase (*spxB*), responsible for converting pyruvate to acetylphosphate, was upregulated 6.28-fold. Similarly, the genes encoding acetic acid kinase (*ackA*), which catalyzes the conversion of acetylphosphate to acetic acid, and lactate-2-oxygenase (E1.13.12.4), which catalyzes the conversion of lactate to acetic acid and CO_2_, were upregulated 6.28-fold and 4.53-fold, respectively, at the gene expression level.

Figures 4C–E demonstrates the gene expression of DEGs enriched in glycolysis, pyruvate metabolism, and oxidative phosphorylation pathways during four sampling points. It shows that genes related to glycolysis and pyruvate metabolism are largely downregulated, while genes related to aerobic respiration are upregulated in *L. paracasei* B1 under low pH conditions (marked with red color). This suggests that *L. paracasei* B1 enhances aerobic respiration at low pH to obtain additional energy to supplement inhibited glycolysis and pyruvate metabolism (Sudawan et al., 2016).

*L. paracasei* B1 is capable of fulfilling its cellular metabolic energy requirements through reversible aerobic respiration. However, this ability comes with a high risk due to the production of reactive oxygen species (ROS) such as hydrogen peroxide and superoxide anion (Maresca et al., 2018). ROS not only attack intracellular molecules, affecting the rate of cell growth, but also have the potential to damage cell structure and genetic information, ultimately leading to cell death (Chen et al., 2023). This may explain the inability to stabilize and the gradual decrease in bacterial density during the late growth phase before the optimization of fermentation culture.

The upregulation of DEGs enriched in the base excision repair pathway confirms the aforementioned observation. During DNA replication, oxidative stress caused by ROS results in various types of DNA damage, including oxidative damage, DNA strand breaks, base modification, and cross-linking damage. These damages severely compromise the integrity and stability of DNA, and the base excision repair mechanism plays a crucial role in repairing DNA damage (Whitaker et al., 2017; Srinivas et al., 2019). In the stationary phase, genes encoding formamidopyrimidine-DNA glycosylase (Fpg), 3-methyladenine-DNA glycosylase (Tag), and DNA polymerase I (DpoI) are upregulated 2.22-fold, 3.77-fold, and 2.83-fold, respectively. These enzymes are responsible for removing and scavenging the modified residues in DNA after DNA damage (Fang et al., 2017). Notably, formamidopyrimidine-DNA glycosylase is one of the key endonucleases involved in the DNA repair process. It catalyzes the excision reaction of gaps or aberrant bases between purine and thymine molecules in DNA induced by oxidants in the bacterium. It is capable of recognizing oxidative damages such as 8-hydroxyguanine and 2,6-dioxoadenine in DNA and excising them from the DNA molecule (Boiteux et al., 1990). Despite the enhanced mechanisms of base excision repair employed by the strains to repair damaged DNA, they were still unable to prevent the decline in bacterial density.

There are 19 differentially expressed genes that are enriched and upregulated in the purine and pyrimidine metabolic pathways, as shown in Figure 4F. These pathways assist in cellular repair of DNA damage for cell survival. Not only do purine and pyrimidine metabolism provide bases for the DNA repair process *per se*, but their intermediary metabolites can also induce and promote DNA repair (Zhou et al., 2020). In purine metabolism, when UV light causes oxidative damage to intracellular guanine dinucleotides, cells can utilize precursor guanosine circuits to enhance guanine deaminase activity and synthetic yield for DNA repair (Cheong and Lee, 2020). Similarly, pyrimidine metabolism can promote DNA repair by regulating the levels of pyrimidine dinucleotides and nucleotides, such as uracil, *in vivo* (Berger et al., 2008).

The 19 downregulated differentially expressed genes in Figure 4F are mainly concentrated in six functional modules: follitropic purine synthesis, follitropic pyrimidine synthesis, guanine ribonucleotide synthesis, pyrimidine deoxyribonucleotide synthesis, pyrimidine deoxyribonucleotide synthesis, and deoxyribonucleotide synthesis. This suggests that, after oxygen stress-induced DNA damage, *L. paracasei* B1 provides DNA repair by enhancing purine and pyrimidine metabolism to provide the required bases and nucleotides for DNA repair. Enhancement of purine and pyrimidine metabolism is very effective in increasing the survival rate of *Lactobacillus*. Additionally, *Lactococcus lactis* can alter its purine metabolism (*deoB, guaA*, and *tktA*) to increase its tolerance to a variety of stresses after heat stress (Gonzalez et al., 2017). Purines have also been used in *Lactobacillus plantarum* processing to attenuate cell membrane and DNA loss, thus improving survival by adding purines (Ma, 2022). Purines and pyrimidines can assist *Lactobacillus* in coping with environmental stress and effectively improve the survival rate of the strain. Furthermore, the previous response pathway to phosphorus limitation can be utilized by adding purines, pyrimidines, and phosphorus-containing inorganic salts as nutrients in the mid- and late-stages of fermentation.

### 3.10 Analysis of enrichment of DEGs between T1 and T2

**FIGURE 5:**
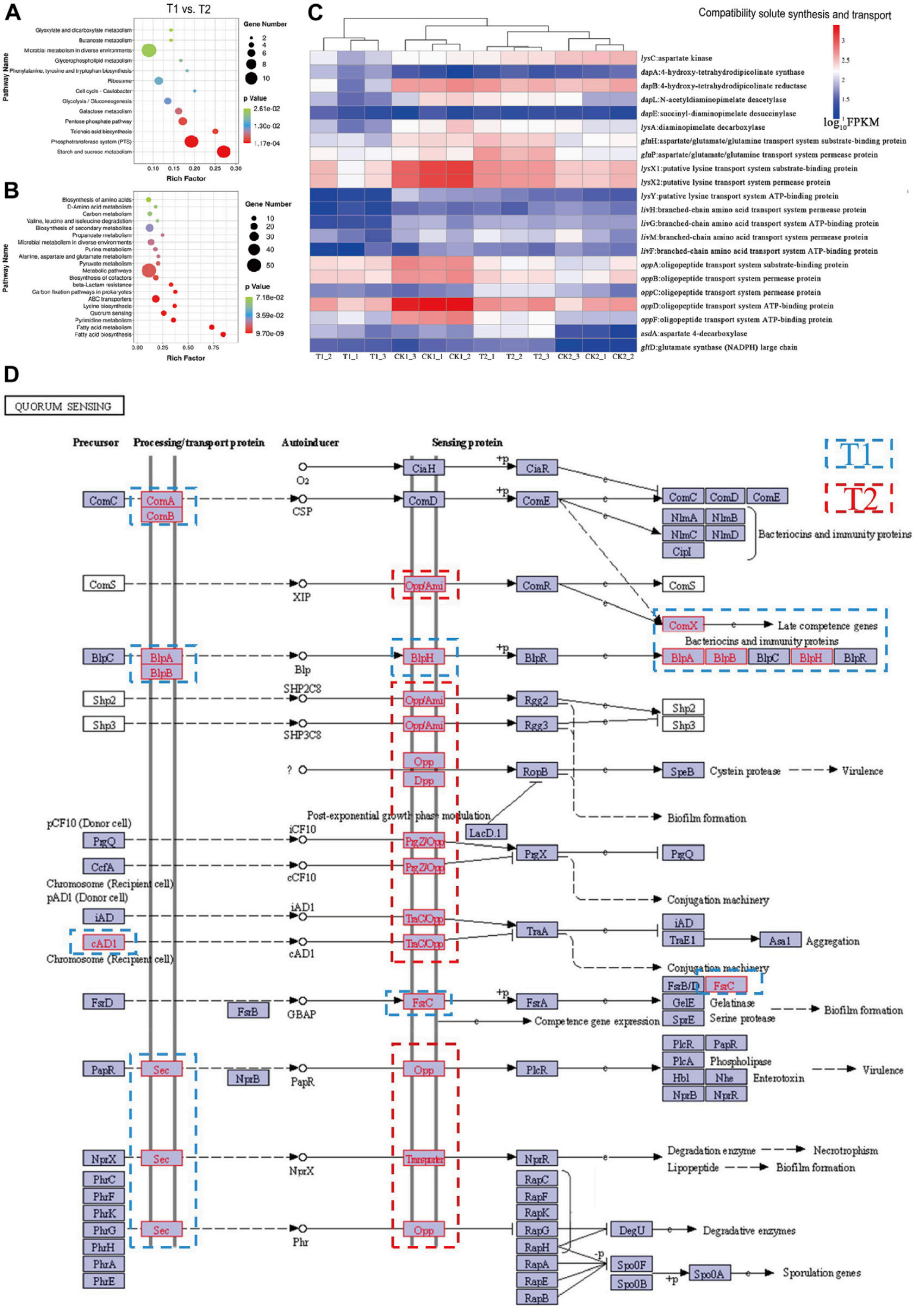
Enrichment bubble diagram of DEGs between T1 vs. T2: **(A)** upregulation, **(B)** downregulation. Abundance of DEGs in pathways: **(C)** compatibility solute synthesis and transport. **(D)** Enrichment annotation of DEGs in quorum sensing (QS) with blue borders representing upregulated genes in the mid-log phase and red borders representing upregulated genes in the stationary phase.

The comparison of between T1 and T2 groups represents the comparison of gene expression between the mid-logarithmic period and the stable period of post-fermentation control optimization (Figure 5A). The differentially expressed genes in this comparison are enriched in carbohydrate metabolism, amino acid metabolism, lipid metabolism, membrane transport, and other pathways.

*L. paracasei* B1 exhibited high activity in various metabolic pathways during the mid-log phase. These pathways included glycolysis, starch and sucrose metabolism, galactose metabolism, and the breakdown of organic acids such as pentose phosphate, glyoxylate, dicarboxylic acid, and butyric acid. This suggests that optimizing the fermentation conditions provided a suitable environment for the strain to efficiently utilize nutrients for growth and metabolism during this stage.

In the logarithmic phase, there was an enrichment of genes related to the Phosphotransferase system (PTS). The PTS system is responsible for the active transport and phosphorylation of sugars in bacteria, and intensifying PTS enhances the uptake of carbon sources, leading to increased carbon fluxes and the synthesis of metabolites (Krahn et al., 2021). Supplementary Figure S6 shows an increase in the expression of ScrA, BglF, CelABC, ManXY, GatABC, and UlaABC genes, indicating enhanced uptake of sugars such as sucrose, cellobiose, mannose, and galactitol via the PTS system. The intensified metabolism of starch and sucrose is influenced by the enhancement of PTS. The stability of PTS is dependent on the cultivation conditions, and studies have shown that low pH conditions negatively impact the phosphotransferase system, reducing enzyme activity, substrate binding capacity, and catalytic efficiency (Wilkins et al., 2002). By controlling fermentation conditions, *L. paracasei* B1 was able to grow at a suitable pH, enhancing the ability of PTS to uptake sucrose, the main sugar source in the medium. This may explain the significant difference in the specific growth rate during the logarithmic period of the two fermentation modes.

PTS phosphorylates and transports sugars across membranes by utilizing the high-energy phosphate group of phosphoenolpyruvate (PEP). PEP is not only an intermediate metabolite of PTS but also a precursor for the synthesis of many amino acids. Therefore, increasing the expression of PEP synthase or redirecting PEP from the amino acid metabolic pathway is an effective way to enhance sugar uptake by strains (Ma et al., 2017). Additionally, microorganisms have a carbon catabolic metabolic mechanism where preferred carbon sources, particularly PTS carbon sources, are utilized first. Different types of PTS carbon sources can have varying effects on strain growth and metabolism. For example, the presence of acetic acid inhibits the expression of the Glc transporter protein in *Corynebacterium glutamicum*, whereas the presence of maltose increases the expression of Glc, thus enhancing glucose utilization by the strains. This highlights the effectiveness of composite carbon sources in medium design for promoting microbial growth (Engels and Wendisch, 2007; Krause et al., 2009). Therefore, using a composite PTS carbon source for medium design could be a potential strategy to optimize strain growth and reduce costs based on *Lactobacillus paracasei*’s translocation sugar preference during the logarithmic phase.

To support rapid growth, *L. paracasei* B1 could bypass acid stress and starvation stress by continuously adding ammonia and nutrient replenishment solution. However, the accumulation of lactic acid ammonium salts formed during acid-base neutralization in the later stages of fermentation would create a high-salt environment, resulting in osmotic pressure stress. Figure 5A illustrates the upregulation of genes involved in amino acid metabolic pathways such as lysine synthesis, alanine metabolism, aspartic acid metabolism, and glutamic acid metabolism. These amino acids have been shown in previous studies to be soluble in water without precipitation or coagulation, and they possess hyperosmotic protective properties (Qu et al., 2012; Gao et al., 2013). Specifically, six differentially expressed genes were enriched in the succinyl I-DAP pathway and acetyl-DAP pathway for lysine synthesis, as shown in Supplementary Figure S7. In addition, in alanine, aspartate, and glutamate metabolism, the genes for aspartate-4-decarboxylase (*asdA*), glutamate synthase (*gltD*), and aspartoacylase (*ansA*) were upregulated, leading to increased synthesis of L-alanine, L-glutamate, and the conversion of L-asparagine to L-aspartic acid. *L*. *paracaseus* B1 upregulates these genes to synthesize and accumulate these amino acids, increasing the intracellular osmotic potential to resist the stress of a hypertonic environment.

*L. paracasei* B1 exhibits limited autonomous synthesis of compatible solutes, relying instead on the ABC transport system for osmoregulation through the uptake of compatible solutes from the environment (Smits et al., 2008). Supplementary Figure S8 demonstrates an upregulation of genes associated with lysine (*lysXY*), branched-chain amino acids (*livFGHM*), oligopeptides (*oppABCDF*), and aspartate and glutamate (*Pev1AB*) transporter systems, which are responsible for encoding receptor proteins, substrate-binding proteins, ATPases, osmolytes, and channel proteins (Doeven et al., 2008). Receptor proteins become activated when the osmotic pressure in the environment surpasses a certain threshold. This activation initiates the expression of corresponding transport system proteins, leading to an enhanced transport of amino acid-compatible solutes to maintain osmotic homeostasis within the organism (Bouvier et al., 2000).

In Figure 5C, the expression distribution of differentially expressed genes related to compatible solute synthesis and transport at the four sampling sites is depicted. The majority of genes exhibited higher expression levels in T2. These results suggest that, after optimizing culture conditions, the bacterium can acquire amino acid-based compatible solutes to alleviate osmotic stress and promote continued growth at a later phase of fermentation. Furthermore, it may be valuable to investigate potential osmoprotective functions of other amino acids, in addition to lysine, glutamate, and aspartic acid that are involved in this pathway. No differentially expressed genes related to DNA repair were enriched in T1 vs. T2, indicating that the optimization of fermentation culture conditions protected *L. paracasei* B1 from acid and oxygen stress damage. Moreover, it suggests that hyperosmotic stress did not cause structural damage to the cell, such as damage to genetic material. The cessation of growth and decrease in density of the bacterium in the stationary phase may be due to quorum sensing (QS). QS is a unique self-regulatory mechanism in which cells communicate through the release of autosensing molecules. This process induces the expression of relevant metabolic genes, regulating behaviors such as biofilm formation, bacteriocin synthesis, and bacterial autolysis (Li et al., 2021). Bacterial density and autoinduction molecules are interconnected. In this study, we assessed the enrichment of differentially expressed genes in quorum sensing during the mid-log phase and stationary phase. The results are shown in Figure 5D, with blue borders representing upregulated genes in the mid-log phase and red borders representing upregulated genes in the stationary phase.

The genes encoding transport proteins, ComAB, BlpAB, and Sec, were upregulated during the logarithmic phase. Conversely, the expression of Opp, Dpp, and transport genes increased upon entering the stationary phase, resulting in the increased translation of sensing proteins and the transmission of signaling molecules from the SHP2C8, SHP3C8, *icF10*, *cad1*, and *phr* populations to downstream target proteins. Notably, NprX, SHP2C8, and SHP3C8 have the ability to induce biofilm formation, suggesting that QS is involved in regulating biofilm formation under possible salt stress to improve bacterial resistance (Wang et al., 2022). Additionally, the signaling pathways mediated by the majority of sensory proteins mainly involve aggregation, genetic material exchange, and cellular decay. These pathways include sensory aggregation, conjugation mechanisms, late transforming genes, activation of degradative enzymes, and necrosis, among others. TCS is likely involved in QS signaling to activate the autolysis response process of the bacteriophage. The expression of the LytR gene of the LytTR family was upregulated 2.09-fold in TCS. This gene encodes the LytT response regulator protein, which contains a structural domain that binds to enzymes related to cell wall structural breakdown. Phosphorylation of the upstream LytS receptor protein enhances the activity of peptidoglycan hydrolases, which sever β-1,4-glycosidic and peptidic bonds, weaken the cell wall, and ultimately lead to cell lysis and bacterial death. This autolysis behavior is distinct from the cellular damage-induced demise observed under unoptimized fermentation processes for *L. paracasei* B1. In this case, the bacterium reduces its density through active autolysis after reaching a certain threshold, leading to self-destruction (Qian et al., 2022). The bacterium releases a signaling molecule during growth, and when its accumulation reaches a certain concentration, it activates the pathway for autolysis, limiting further bacterial reproduction. This behavior allows the colony to continue in complex survival environments at the expense of some individuals but restricts further biomass increase in industrial production settings.

To overcome the limitation of bacterial reproduction by blocking autolysis signaling in population sensing, methods such as gene modification and knockout of signaling systems and cellular autolysis mechanisms can be employed. However, this approach requires balancing the impairment of cellular physiological functions and biomass accumulation (Liu et al., 2021). Additionally, the use of QS signaling inhibitors to degrade QS signaling or block binding to signaling receptors generally does not affect microbial growth. This may be a strategy for *Lactobacillus paracasei* to further enhance biomass at high densities (Pang et al., 2016).

#### 3.10.1 Enrichment analysis of differentially expressed genes in CK1 and T1

**FIGURE 6:**
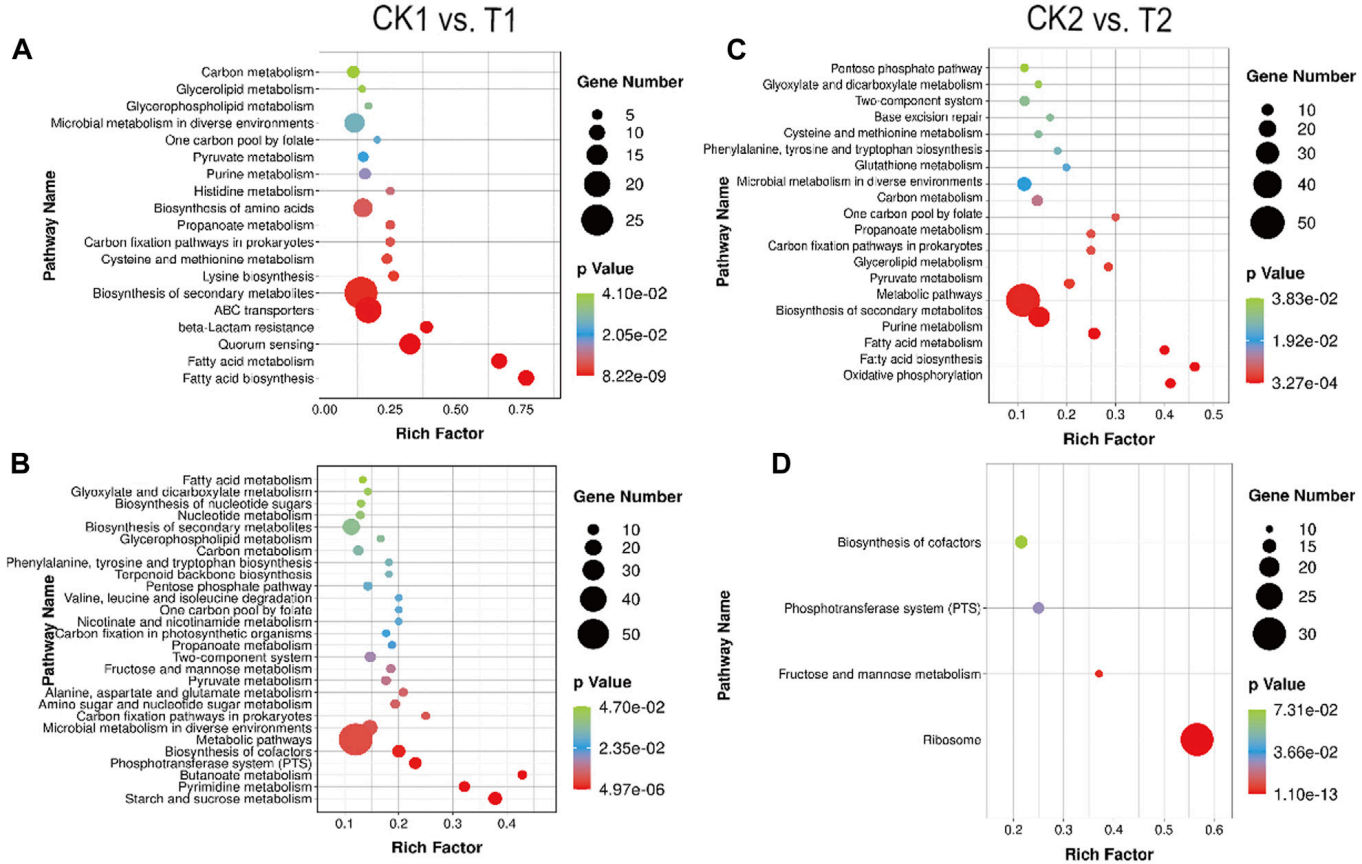
Enrichment bubble diagram of DEGs between CK1 vs. T1: **(A)** upregulation, **(B)** downregulation. Enrichment bubble diagram of DEGs between CK2 vs. T2: **(C)** upregulation, **(D)** downregulation.

Figures 6A, B displays the comparison between the CK1 and T1 groups, which represent the logarithmic mid-phase period in both fermentation modes. By controlling temperature, pH, and substrate sugar concentration to create an optimal growth environment for *L. paracasei* B1, the sugar uptake capacity of T1 was increased compared to CK1. Specifically, the expression of ScrA, BglF, and MtlA encoding genes in the PTS system was upregulated by 3.14-fold, 4.51-fold, and 2.29-fold, respectively, in T1 relative to CK1. Proteins are transported to the cells via the PTS system and play a crucial role in synthesizing molecules that are compatible with their own energy production mechanism, thereby providing an ample supply of carbon sources for the fermentation process (Huang et al., 2020). Furthermore, the optimization of fermentation led to an enhancement in the uptake capacity of sucrose, β-glucoside, and mannitol by *L. paracasei* B1. This further supports the conclusions and analysis results mentioned previously. Additionally, the gene encoding SrlABE responsible for sorbitol transport was upregulated by 2.11-fold, 2.35-fold, and 2.25-fold in CK1. Given that sorbitol is commonly used as a protective agent in lactic acid bacteria processing, this study proposes that the increased uptake of sorbitol is an adaptive response to an adverse environment (Ambros et al., 2018).

The optimization of culture conditions in T1 not only reduced environmental stress but also facilitated enhanced nutrient translocation. As a result, more abundant material and energy metabolic pathways were expressed in T1. These included pathways related to carbohydrate metabolism (starch and sucrose metabolism, fructose and mannose metabolism), amino acid metabolism (aspartate and glutamate metabolism), nucleotide metabolism (nicotinate and nicotinamide metabolism), as well as higher levels of pyruvate metabolism and glycolytic metabolism. These enhancements in nutrient intake and energy metabolism promoted faster growth and proliferation in *L. paracasei* B1, ultimately leading to high-density levels. Additionally, CK1 exhibited unique metabolic enrichments. Genes related to fatty acid synthesis and metabolism, lysine synthesis, cysteine and methionine metabolism, histidine metabolism, and the glycerophospholipid pathway were significantly upregulated in CK1.

Of interest, the expression of the LuxS gene, involved in quorum sensing (QS), was increased 2.01-fold in CK1. The corresponding LuxS signaling pathway is depicted in Supplementary Figure S9. The LuxS gene encodes the population-sensing molecule AI-2, which then activates the transcription of downstream target genes (Dai, 2018). Previous studies have demonstrated that when subjected to acid stress, *Lactobacillus* thermophilus can adapt to the acidic environment by upregulating transcription of the LuxS gene, enhancing AI-2 activity and cellular biofilm synthesis. Consequently, the LuxS/AI-2 response also regulates the growth and metabolic processes of *L. paracasei* B1 in response to environmental stress (Azcarate-Peril et al., 2005). Activation of the LuxS/AI-2 response is dependent on specific environmental factors. In several studies investigating salt tolerance in lactic acid bacteria, it was observed that AI-2 activity varied among different strains based on salt concentration. This variability was attributed to differences in stability of AI-2 activity and strain-specific osmotic pressure tolerance and the existence of osmotic pressure tolerance differences among strains (Yeo et al., 2015).

In summary, the QS system plays a significant role in how *L. paracasei* B1 responds to environmental stress during fermentation, as well as in regulating bacterial density adaptation. The precise regulation of the QS system can be beneficial in controlling the growth process of these strains in industrial production.

#### 3.10.2 Enrichment analysis of differentially expressed genes in CK2 vs. T2

Comparison of CK2 and T2 groups revealed differences in the stabilization periods of two distinct fermentation modes. Figures 6C, D depicts that CK2 exhibits a greater number of upregulated DEGs in comparison to T2. These DEGs are primarily enriched in metabolic pathways induced by acid stress and oxidative stress.

It is evident that acid stress disrupts the stable environment necessary for glycolysis (Desriac et al., 2013). *L. paracasei* B1 compensates for this disruption by generating additional energy through aerobic respiration. This ensures the efficient operation of the proton pump and helps maintain the stability of the intracellular environment. As a result, reactive oxygen clusters, such as H_2_O_2_, are generated, which can attack cellular structures. Furthermore, as illustrated in Figure 5C, *L. paracasei* B1 not only participates in purine and pyrimidine metabolism, base excision repair, and other pathways, but also contributes to defenses against oxidative damage through the thioredoxin and glutaredoxin systems. These defense mechanisms involve glutathione metabolism, phenylalanine, tyrosine, and tryptophan synthesis, and cysteine and methionine metabolism (Lu and Holmgren, 2014). Specific genes encoding cysteine synthase (*cysK*) and glutathione reductase (*gor*) were found to be upregulated by 4.03-fold and 2.99-fold, respectively. Cysteine and methionine are thioredoxin-reducing proteins with antioxidant properties. They serve as targets for reactive oxygen clusters, reducing the production of other oxygen byproducts in the cell through the sulfur/disulfide balance. Calderini et al. demonstrated that *Lactobacillus acidophilus* NCFM maintains cellular stability by enhancing cysteine synthesis and increasing the ratio of intracellular reductase (Calderini et al., 2017). Glutathione (GSH), an abundant low molecular weight antioxidant in the cell, also plays a role in protecting cellular stability by trapping free radicals. Oxidized glutathione (GSSG) can be reduced back to GSH through the catalysis of glutathione reductase, utilizing NADPH. This process scavenges reactive oxygen clusters within the cell (Kalinina et al., 2008). It has been observed that media culture of lactic acid bacteria with moderate amounts of GSH significantly improves indicators such as strain activity and antioxidant capacity of *Lactobacillus*, indicating the positive effect of GSH on bacterial growth and metabolism (Zhang, 2008). Besides, it was shown that *Lactobacillus bulgaricus* and *Streptococcus thermophilus* obtain the CysE gene and other genes through the process of transformation. This acquisition results in an increase in the levels of methionine and cysteamine, while also providing a carbon source for co-fermentation and promotes the acidification effect during the fermentation of yogurt (Liu et al., 2009). In this study, the upregulation of enzymes related to cysteine synthesis and glutathione conversion reflects the need for both during the alleviation of oxygen stress by *L. paracasei* B1. Therefore, adding sulfur-containing amino acid substrates like cysteine and glutamate to the medium or fermentation broth can enhance the thioredoxin system and glutaredoxin system, increasing cellular adaptability to oxygen stress. This enhancement can improve the survival and activity of the strain during fermentation production and subsequent strain preservation.

### 3.11 Evolutionary inference of the differentially expressed genes

The term “mobileome” refers to gene profiles in the microbial genome that have been acquired through horizontal gene transfer (HGT) (Choi and Kim, 2007). We aim to determine if the aforementioned DEGs were affected by the HGT process. To achieve this, we mapped the sequences of the DEGs onto the identified “mobileome” of Lactobacillaceae (as described in the Method section). A total of 57 DEG items were found to match the mobileome of Lactobacillaceae. These items include the following categories (Supplementary Table S5).

1) DEGs involved in carbohydrate and energy metabolism, which were found to be significantly expressed in CK1 and T1. Examples include 6-phospho-beta-glucosidase, acquired from *Holdemania*, acetolactate synthase, acquired from *Clostridium*, cytochrome d oxidase subunit CydB, acquired from *Nakamurella*, NADPH:quinone reductase, acquired from *Rhodococcus*, and phospholipase, acquired from Peptoniphilus.
2) DEGs related to membrane transport, which were found to be significantly expressed in T1 and T2. Examples include PTS system IIC component, acquired from *Holdemanella*, and polyamine/organocation transporter, acquired from *Staphylococcus.*
3) DEGs involved in replication and repair, which were found to be significantly expressed in CK2. Examples include excinuclease ABC subunit A, acquired from *Clostridium*, and recombinational DNA repair ATPase RecF, acquired from *Vallitalea.*
4) DEGs related to translation, which were found to be significantly expressed in CK1. An example is SSU ribosomal protein S18P.
5) DEGs of transcriptional regulators, such as LacI.
6) DEGs involved in signal transduction systems, such as (p)ppGpp synthase.

These findings suggest that HGT may play a role in the domestication and adaptive evolution of fermented microorganisms, such as *Lactobacillus*. This, in turn, contributes to improved fermentation processes, which aligns with previous research (Wang et al., 2023). The occurrence of HGT in a specific environment is advantageous for the domestication of starter cultures and plays an important role in the quality of fermentation (Gibbons and Rinker, 2015; Legras et al., 2018). For instance, previous studies have shown that *Lactobacillus* spp. horizontally acquired genes that enable the development of specific metabolic functions, including nucleoside scavenging, catabolism of arginine, the formation of biofilms and the ability to adapt to alterations in redox and oxygen levels. This acquisition of specialized genes contributes to the extension of shelf life in fermented environments (Nawaz et al., 2011; Nyquist et al., 2011).

## 4 Conclusion

The current study investigates the transcriptome characteristics of *L. paracasei* B1 samples. Transcriptome sequencing was conducted to analyze the samples at different growth stages within the same fermentation mode and between different fermentation modes at the same growth stage. Ultimately, this research can contribute to the further development and utilization of the production capabilities of *L. paracasei* B1.

The results unveiled distinct gene expression patterns among the samples. Specifically, the samples obtained from the experimental group, characterized by unoptimized fermentation culture conditions, demonstrated the weakest correlation between the samples collected at the stationary phase sampling point and those obtained at the other three sampling points. However, there was a higher correlation between the samples from the middle of the logarithmic phase under the two fermentation modes. Additionally, the correlation between the mid-log phase samples under the two fermentation modes was higher compared to the other three samples, whereas the correlation between the mid-log phase samples and the stationary phase samples under the same fermentation mode was lower.

Furthermore, the current study identified the DEGs between the mid-log phase and stationary phase samples. Before the optimization of the non-fermented culture conditions, 627 DEGs were found between the mid-log phase and stationary phase samples (CK1 vs. CK2). After the optimization, 607 DEGs were identified between the mid-log phase and stationary phase samples (T1 vs. T2). Moreover, there were 507 DEGs between CK1 vs. T1 and 671 DEGs between CK2 vs. T2.

This study has found the optimization of fermentation culture conditions for *L. paracasei* B1 has been shown to provide a suitable growth environment, leading to enhanced glycolysis and pyruvate metabolism. The strain’s ability to generate additional energy through aerobic respiration and protect against acid and oxygen stresses has also been highlighted. Fermentation optimization has facilitated rapid growth and accumulation of amino acid-compatible solutes, while the QS system has played a role in adaptation to environmental stress and regulation of bacterial density.

The results of the KEGG pathway-based enrichment analysis revealed that the optimized fermentation culture conditions created an optimal growth environment for *L. paracasei* B1. Under these conditions, the strain upregulated the expression of genes encoding ScrA, BglF, and MtlA in the PTS system. Consequently, there was an enhanced uptake of sucrose, the main sugar source in the medium, as well as other sugar sources like mannose. This led to the maintenance of high levels of glycolysis and pyruvate metabolism, resulting in rapid growth and reproduction. This intrinsic mechanism is responsible for driving the culture level of *L. paracasei* B1 to reach a high density.

Furthermore, acidity stress negatively impacted glycolysis and pyruvate metabolism. Hence, *L. paracasei* B1 possesses a range of genes (*cydAB*, etc.) that encode electron transport chain-related genes. These genes enable the strain to generate additional energy through aerobic respiration, supporting energy metabolism. However, aerobic respiration also leads to oxygen stress, which can damage cells. To counteract this, the strain enhances glutathione metabolism, cysteine and methionine metabolism, base excision repair, and purine and pyrimidine metabolism to withstand the oxygen stress caused by mixed respiration.

Fermentation optimization allows *L. paracasei* B1 to grow rapidly, free from acid and oxygen stress damage to the organism. During the growth process, the strain accumulates amino acid compatible solutes by upregulating lysine synthesis, alanine, aspartic acid, and glutamic acid metabolism pathways. Additionally, it relies on the ABC transporter system to resist osmotic stress resulting from acid-base neutralization.

## Data Availability

The transcriptome raw data have been make available in the NCBI Sequence Read Archive (SRA) database at https://www.ncbi.nlm.nih.gov/sra/PRJNA1020974. The genome assembly of *Lactobacillus paracasei* is available at https://ngdc.cncb.ac.cn/bioproject/browse/PRJCA020011.
