# Supplementary material for: Transcriptomics of Lactobacillus paracasei: metabolism patterns and cellular responses under high-density culture conditions
Source: Front Bioeng Biotechnol. 2023 Oct 12;11:1274020. doi: 10.3389/fbioe.2023.1274020 (PMC10601642; doi:10.3389/fbioe.2023.1274020)
Supplement: Supplementary file 1 [file DataSheet1.docx]

Supplementary Materials

TableS1 Total RNA purity and concentration detection

| Sample | OD260/280 | OD260/230 | RNA content (µg/uL) | RNA amount（µg） |
| --- | --- | --- | --- | --- |
| CK2-3 | 1.95 | 1.55 | 0.03 | 1.35 |
| T1-1 | 2.43 | 1.28 | 0.02 | 0.92 |
| T1-2 | 2.16 | 2.04 | 0.04 | 1.74 |
| T1-3 | 2.18 | 1.63 | 0.02 | 0.94 |
| T2-1 | 1.90 | 1.53 | 0.06 | 2.53 |
| T2-2 | 1.93 | 2.02 | 0.08 | 3.11 |
| T2-3 | 1.87 | 2.04 | 0.08 | 3.30 |


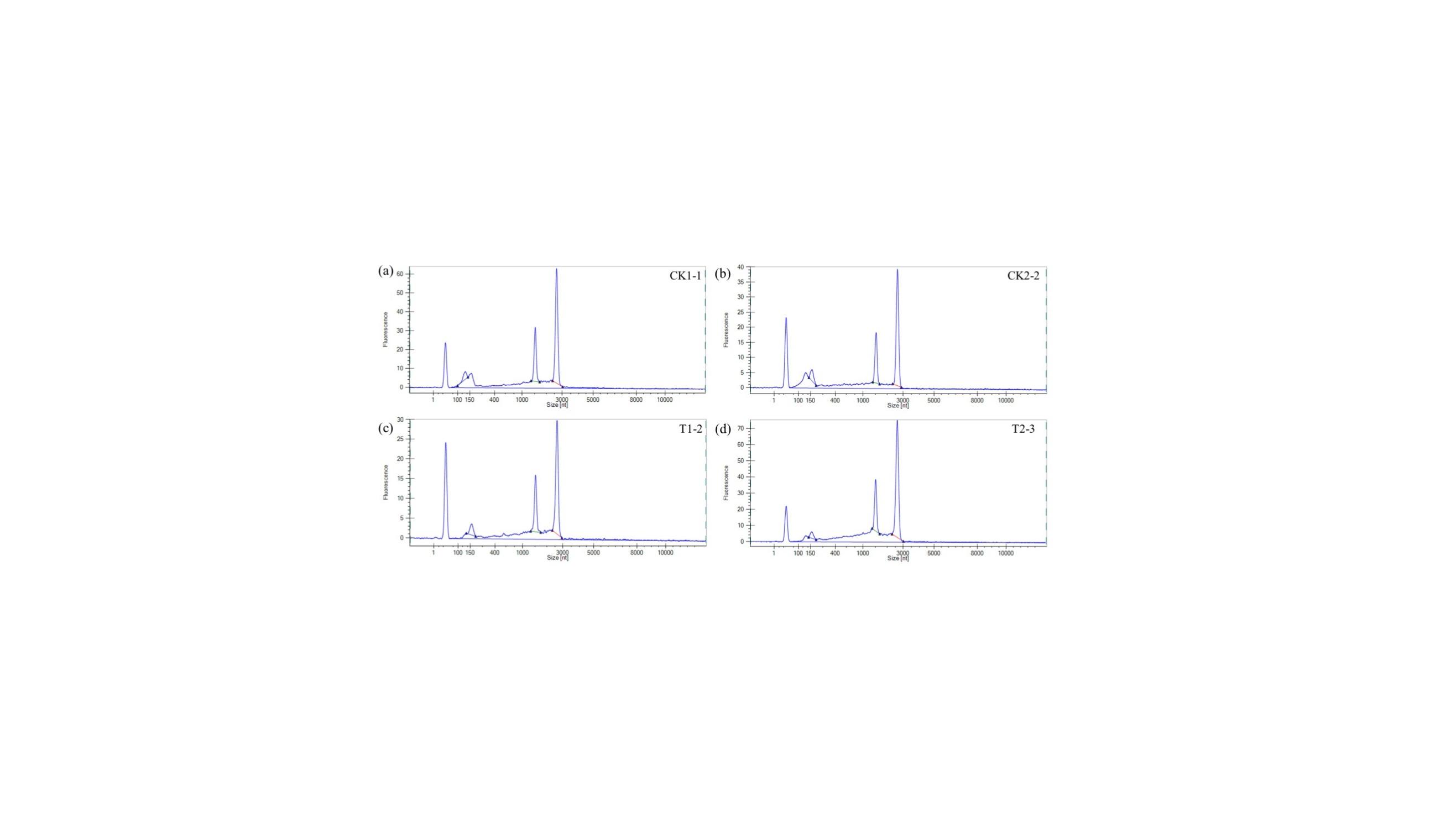


Figure S1 RNA quality peak of random samples

Table S2 sample sequencing quality assessment

| Sample | Clean reads | Q20(%) | Q30(%) | GC(%) | Error rate |
| --- | --- | --- | --- | --- | --- |
| CK1-1 | 4376990 | 97.24 | 92.29 | 46.92 | 0.01 |
| CK1-2 | 3177963 | 97.76 | 93.45 | 47.05 | 0.01 |

| CK1-3 | 3632547 | 97.75 | 93.45 | 46.81 | 0.01 |
| --- | --- | --- | --- | --- | --- |
| CK2-1 | 4137093 | 97.35 | 92.56 | 47.51 | 0.01 |
| CK2-2 | 4597265 | 97.80 | 93.52 | 47.04 | 0.01 |
| CK2-3 | 4945052 | 97.01 | 91.79 | 47.63 | 0.01 |
| T1-1 | 4509786 | 97.47 | 92.89 | 46.62 | 0.01 |
| T1-2 | 4024512 | 97.67 | 93.34 | 46.82 | 0.01 |
| T1-3 | 3630158 | 97.98 | 94.05 | 46.41 | 0.01 |
| T2-1 | 3990719 | 96.97 | 92.06 | 47.79 | 0.01 |
| T2-2 | 4301515 | 97.68 | 93.31 | 47.29 | 0.01 |
| T2-3 | 3946948 | 97.60 | 93.20 | 47.51 | 0.01 |

Table S3 Sequence alignment results with L*actobacillus paracasei* B1 genome

| Sample | Input reads | Uniquely mapped reads | Uniquely mapped reads | Unmapped reads | Unmapped reads ratio |
| --- | --- | --- | --- | --- | --- |
| CK1-1 | 4376990 | 4166925 | 95.20% | 4.80% | 4.80% |
| CK1-2 | 3177963 | 3004930 | 94.56% | 5.44% | 5.44% |

| CK1-3 | 3632547 | 3422504 | 94.22% | 5.78% | 5.78% |
| --- | --- | --- | --- | --- | --- |
| CK2-1 | 4137093 | 3640671 | 88.00% | 12.00% | 12.00% |
| CK2-2 | 4597265 | 4299084 | 93.51% | 6.49% | 6.49% |
| CK2-3 | 4945052 | 8480969 | 94.81% | 5.19% | 5.19% |
| T1-1 | 4509786 | 4325017 | 95.90% | 4.10% | 4.10% |
| T1-2 | 4024512 | 3852905 | 95.74% | 4.26% | 4.26% |
| T1-3 | 3630158 | 3497903 | 96.36% | 3.64% | 3.64% |
| T2-1 | 3990719 | 3792779 | 95.04% | 4.96% | 4.96% |
| T2-2 | 4301515 | 4135607 | 96.14% | 3.86% | 3.86% |
| T2-3 | 3946948 | 3794536 | 96.14% | 3.86% | 3.86% |

Table S4 Statistics of the number of genes in different expression levels

| CK2-2 | 51(1.74%) | 42(1.44%) | 282(9.64%) | 759(25.96%) | 1790(61.22%) |
| --- | --- | --- | --- | --- | --- |
| CK2-3 | 107(3.58%) | 54(1.8%) | 343(11.46%) | 752(25.13%) | 1737(58.04%) |
| T1-1 | 57(1.94%) | 51(1.73%) | 239(8.12%) | 815(27.67%) | 1783(60.54%) |
| T1-2 | 64(2.17%) | 33(1.12%) | 186(6.29%) | 734(24.84%) | 1938(65.58%) |
| T1-3 | 59(2.01%) | 36(1.23%) | 221(7.53%) | 818(27.87%) | 1801(61.36%) |
| T2-1 | 77(2.58%) | 65(2.18%) | 284(9.53%) | 681(22.85%) | 1873(62.85%) |
| T2-2 | 71(2.43%) | 44(1.51%) | 262(8.98%) | 718(24.6%) | 1824(62.49%) |
| T2-3 | 60(2.03%) | 53(1.8%) | 262(8.88%) | 700(23.73%) | 1875(63.56%) |


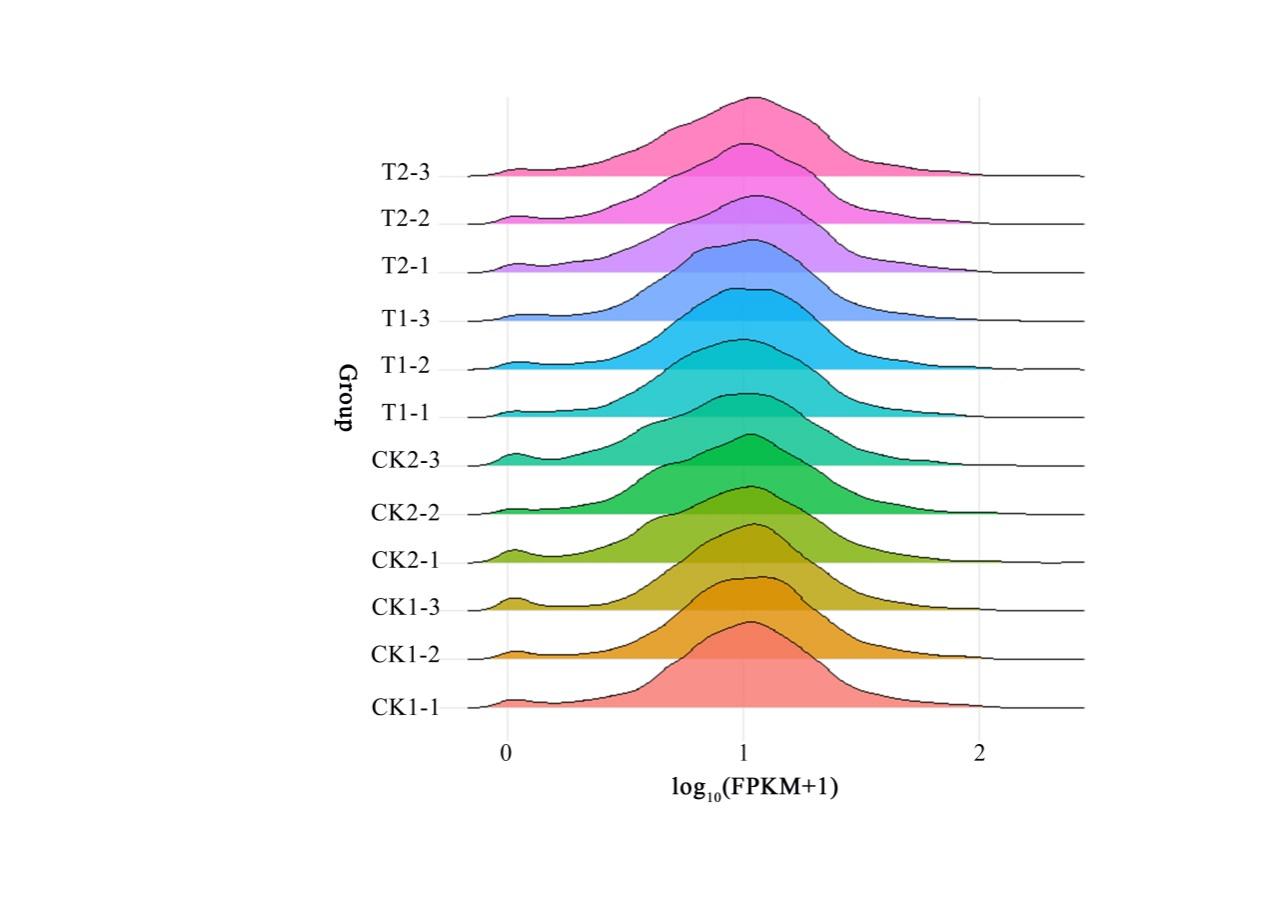


| Sample | FPKM(0-1) | FPKM(1-3) | FPKM(3-15) | FPKM(15-60) | FPKM(＞60) |
| --- | --- | --- | --- | --- | --- |
| CK1-1 | 70(2.37%) | 39(1.32%) | 172(5.83%) | 727(24.65%) | 1941(65.82%) |
| CK1-2 | 66(2.27%) | 30(1.03%) | 165(5.69%) | 698(24.05%) | 1943(66.95%) |
| CK1-3 | 103(3.64%) | 36(1.27%) | 147(5.19%) | 701(24.77%) | 1843(65.12%) |
| CK2-1 | 113(3.77%) | 43(1.43%) | 302(10.07%) | 746(24.87%) | 1795(59.85%) |

Figure S2 Gene expression density distribution


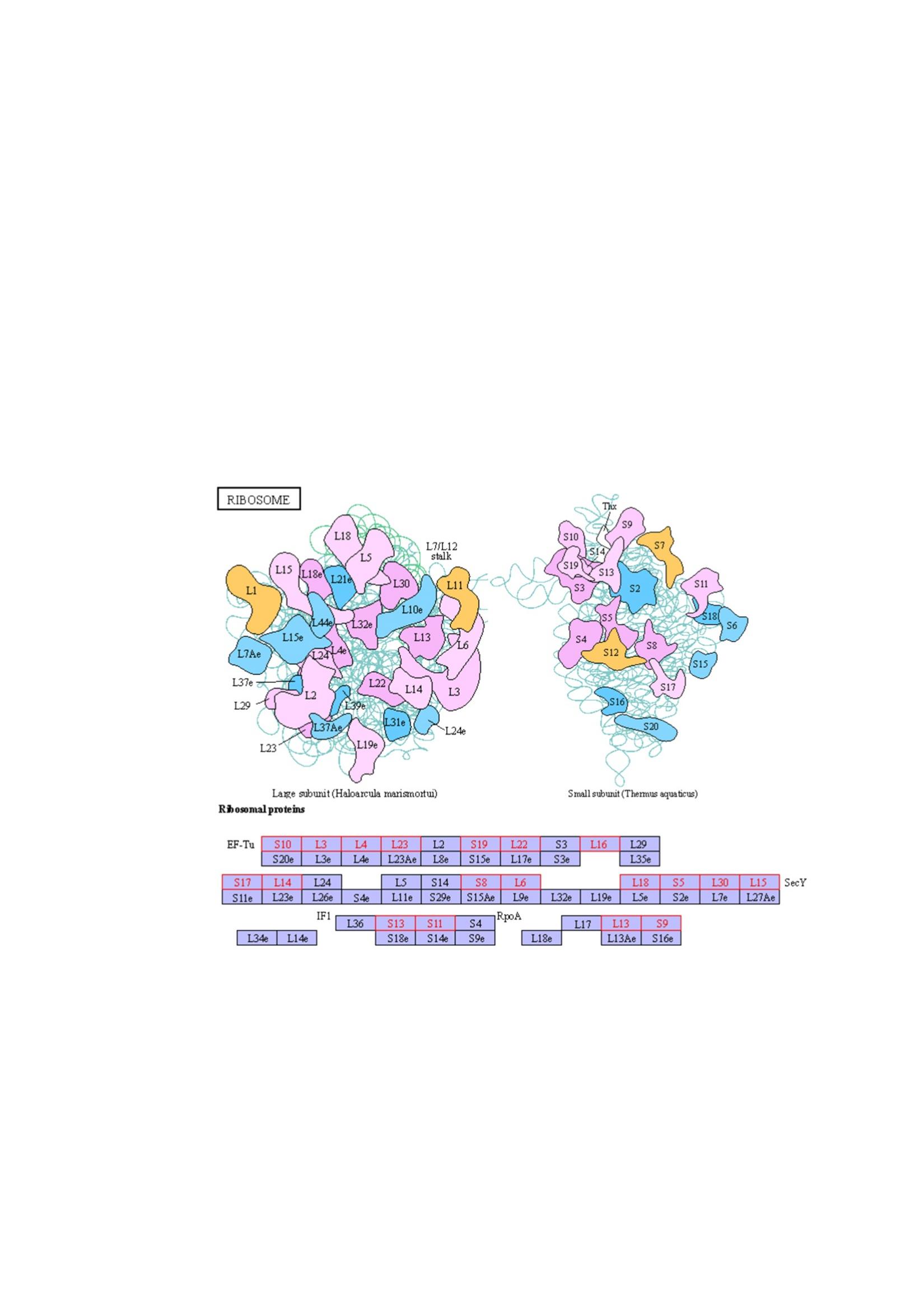


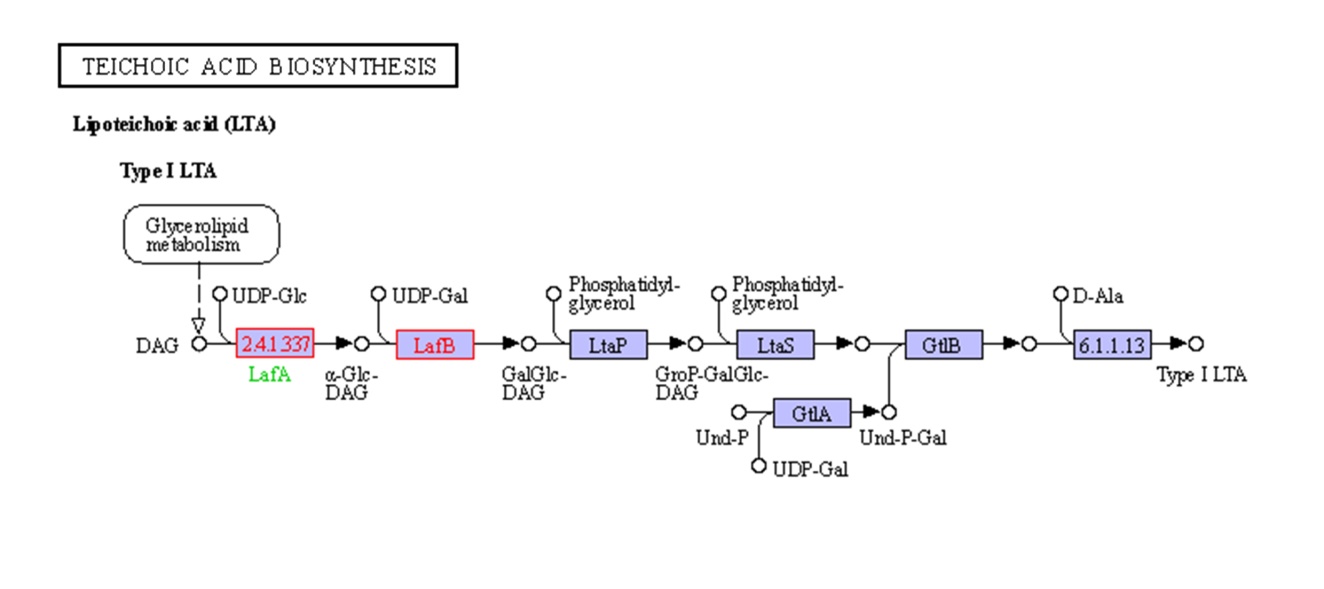
Figure S3 Enrichment annotation of ribosomal protein-related differentially expressed genes. Note: red markers indicate that the gene is a differentially altered gene in the comparison group.

Figure S4 Enrichment annotation of DEGs in teichoic acid biosynthesis


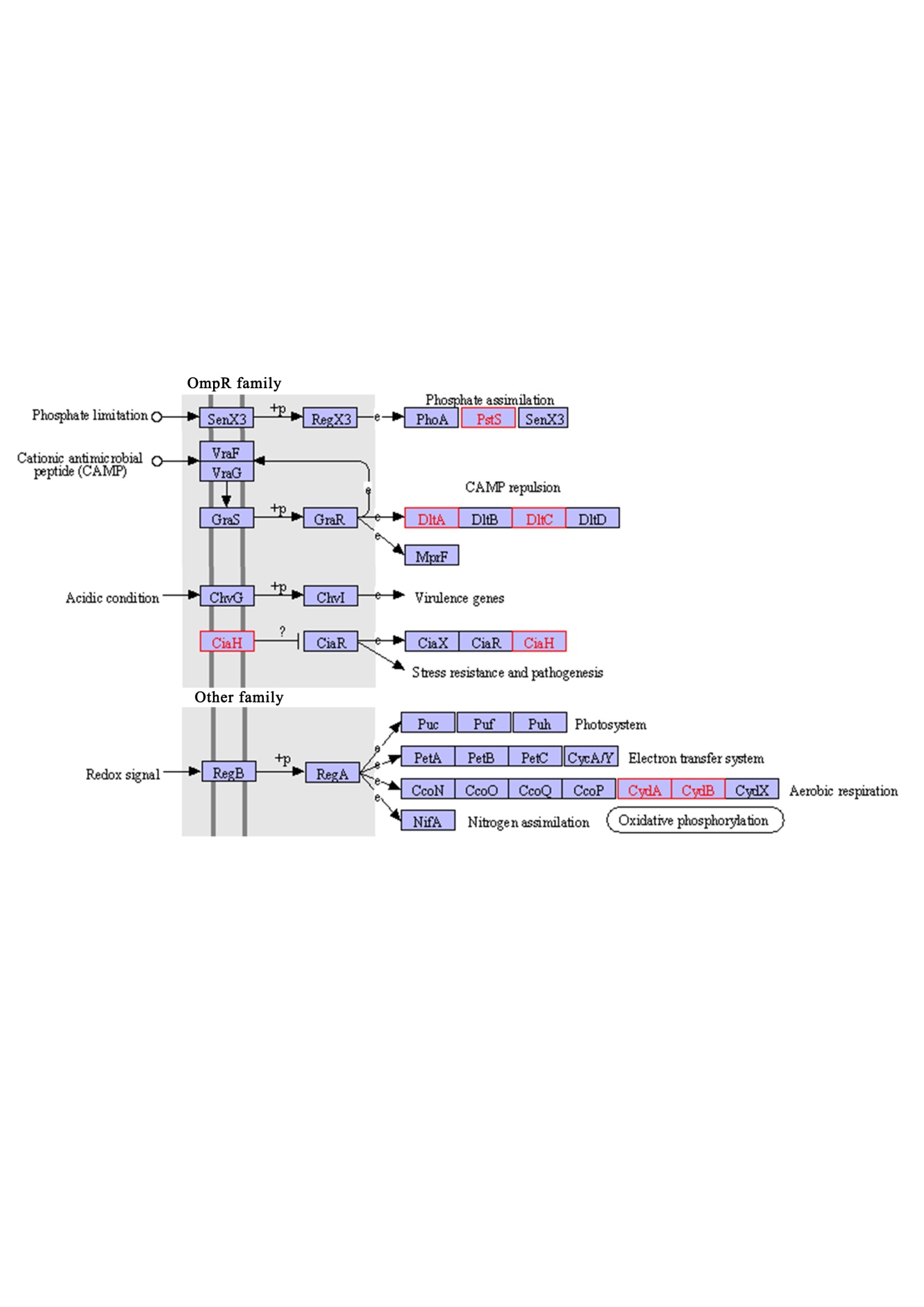


Figure S5 Enrichment annotation of DEGs in two-component system (TCS).


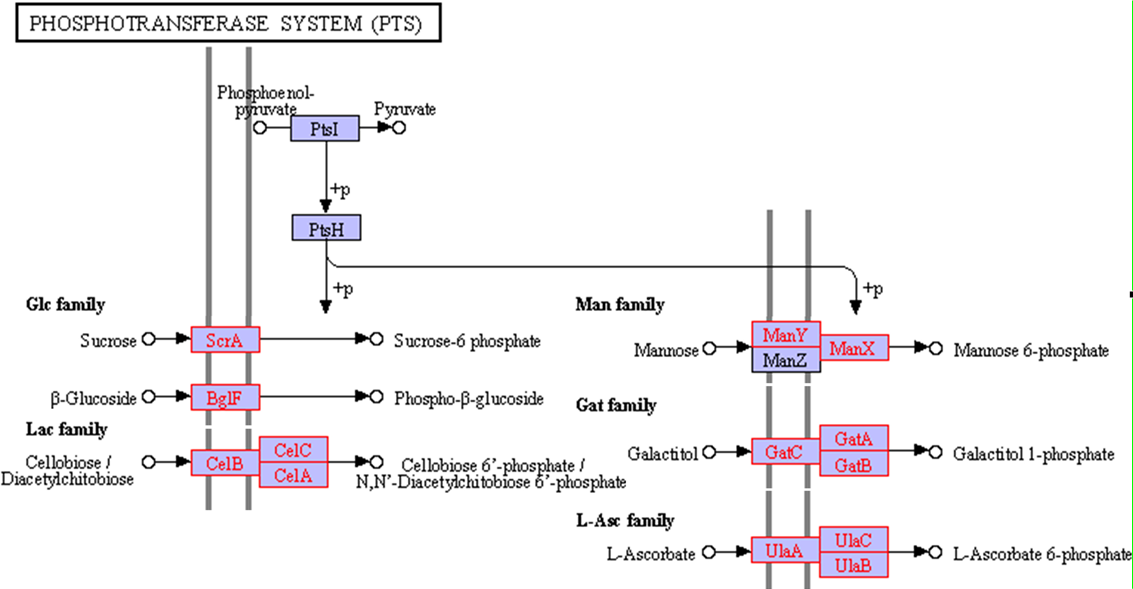


Figure S6 Enrichment annotation of DEGs in PTS system.


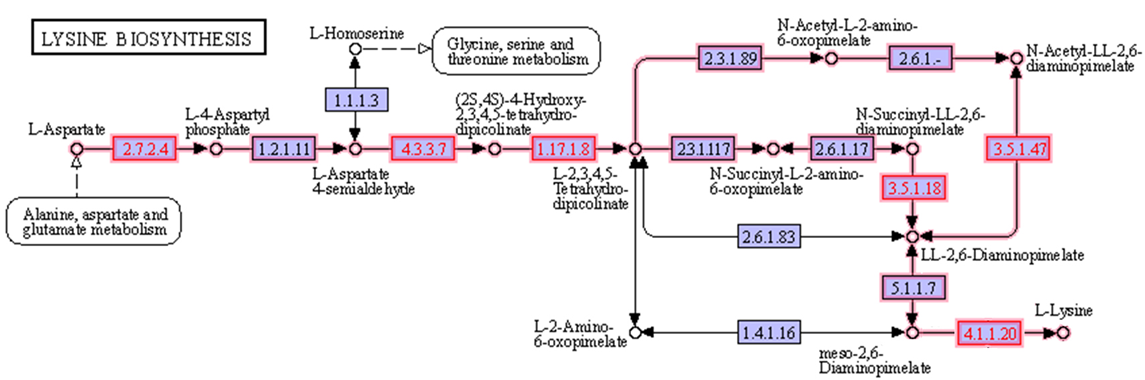


Figure S7 Enrichment annotation of DEGs in lysine synthesis pathway


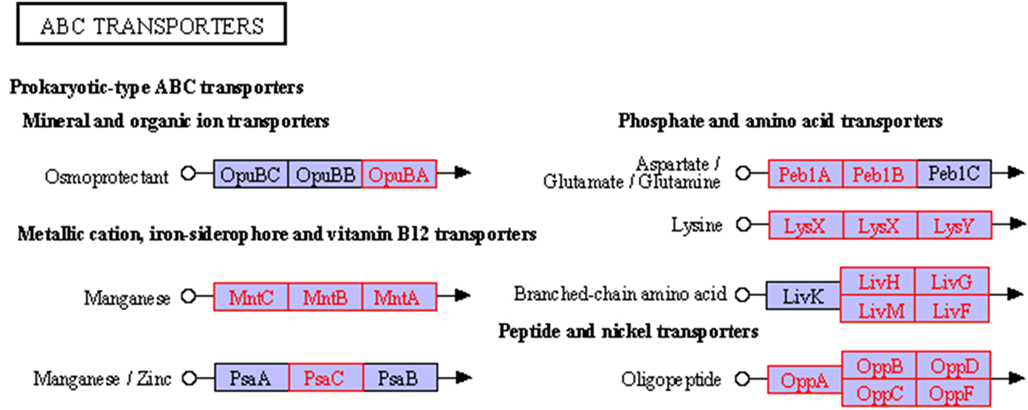


Figure S8 Enrichment annotation of DEGs in ABC transporter system.


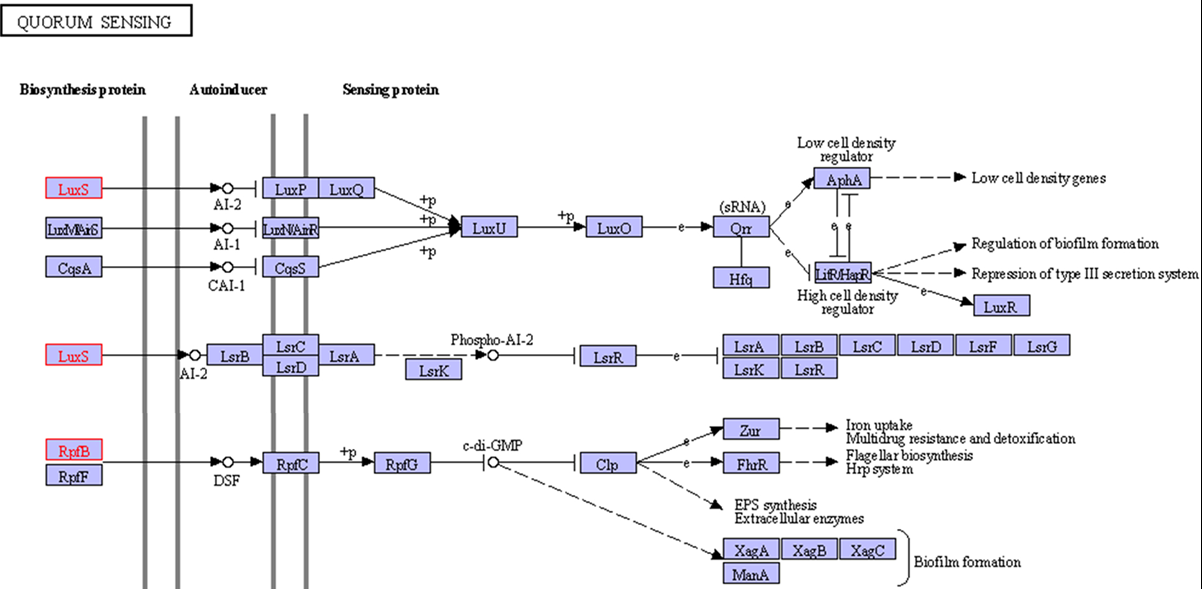


Figure S9 Enrichment annotation of DEGs in quorum sensing.

Table S5 DEGs in *L. paracasei* putatively affected by the HGT process

| Differentially expressed gene in this study | Representative recipient gene sequence (IMG accession) | Gene function | Representative donor gene sequence (IMG accession) | Representative donor |
| --- | --- | --- | --- | --- |
| CAGKPHEN_02102 | 2882297973 | transposase-like protein | 2708693792 | Lactobacillus phage CL1 |
| CAGKPHEN_00115 | 8004847357 | 2,3-bisphosphoglycerate-dependent phosphoglycerate mutase | 2938733054 | Lactobacillus acidophilus NCTC13720 |
| CAGKPHEN_00702 | 2510425744 | **6-phospho-beta-glucosidase** | 644158790 | Holdemania filiformis VPI J1-31B-1, DSM 12042 |
| CAGKPHEN_02828 | 2683114091 | **acetolactate synthase, large subunit** | 2651192699 | Clostridium sp. IODB-O3 |
| CAGKPHEN_01082 | 2683114135 | D-3-phosphoglycerate dehydrogenase | 2651192656 | Clostridium sp. IODB-O3 |
| CAGKPHEN_01910 | 8004847798 | glyceraldehyde 3-phosphate dehydrogenase | 2872423816 | Lactobacillus johnsonii UMNLJ22 |
| CAGKPHEN_01574 | 2683113869 | UTP-hexose-1-phosphate uridylyltransferase | 2651191502 | Clostridium sp. IODB-O3 |
| CAGKPHEN_00182 | 2977623633 | **cytochrome d oxidase subunit CydB** | 645040971 | Nakamurella multipartita Y-104, DSM 44233 |
| CAGKPHEN_02521 | 2967887274 | **(p)ppGpp synthase/HD superfamily hydrolase** | 2898896034 | Brevibacillus dissolubilis CHY01 |
| CAGKPHEN_00265 | 2683114151 | **amidase** | 2651195767 | Clostridium sp. IODB-O3 |
| CAGKPHEN_02541 | 2664584765 | PRD domain-containing protein | 2629577457 | Oceanobacillus oncorhynchi |
| CAGKPHEN_00481 | 2967826355 | DNA-directed RNA polymerase beta subunit | 2555470869 | Bacillus safensis VK |
| CAGKPHEN_00445 | 8002250644 | DNA-directed RNA polymerase subunit alpha | 2933004731 | Liquorilactobacillus nagelii AGA58 |
| CAGKPHEN_02003 | 2964752095 | **aspartyl/glutamyl-tRNA(Asn/Gln) amidotransferase B subunit** | 2507132643 | Thiomicrospira aerophila AL3 |
| CAGKPHEN_02493 | 2683113718 | glycyl-tRNA synthetase beta chain | 2651192845 | Clostridium sp. IODB-O3 |
| CAGKPHEN_00913 | 8004850315 | SSU ribosomal protein S18P | 2539549788 | Ligilactobacillus ruminis ATCC 25644 |
| CAGKPHEN_02247 | 2967804537 | valyl-tRNA synthetase | 2757442856 | Petrotoga olearia DSM 13574 |
| CAGKPHEN_01868 | 2683114374 | **ribonucrease Y** | 2651192945 | Clostridium sp. IODB-O3 |
| CAGKPHEN_01900 | 2683114192 | **excinuclease ABC subunit A** | 2651191449 | Clostridium sp. IODB-O3 |
| CAGKPHEN_00610 | 2683114375 | **recombination protein RecA** | 2651192946 | Clostridium sp. IODB-O3 |
| CAGKPHEN_00906 | 2967871476 | **recombinational DNA repair ATPase RecF** | 2887391146 | Vallitalea guaymasensis L81 |
| CAGKPHEN_00704 | 2510425746 | **PTS system beta-glucosides-specific IIC component** | 643184134 | Holdemanella biformis DSM 3989 |
| CAGKPHEN_01707 | 2683114346 | Xaa-Pro dipeptidase | 2651192918 | Clostridium sp. IODB-O3 |
| CAGKPHEN_02170 | 2967885605 | actin-like ATPase involved in cell morphogenesis | 2789210696 | Traorella massiliensis Marseille-P3110 |
| CAGKPHEN_00908 | 2970241759 | DNA gyrase subunit A | 2559027994 | Haploplasma modicum ATCC 29102 |
| CAGKPHEN_02403 | 2957660980 | DNA gyrase/topoisomerase IV subunit A | 2640650407 | Spiroplasma atrichopogonis GNAT3597 |
| CAGKPHEN_00703 | 2510425745 | **LacI family transcriptional regulator** | 650074205 | Coprobacillus cateniformis 29_1 |
| CAGKPHEN_02902 | 2518702149 | phage replication initiation protein | 641809926 | Dorea formicigenerans ATCC 27755 |
| CAGKPHEN_01393 | 8002251462 | Transposase InsO and inactivated derivatives | 2673352055 | Liquorilactobacillus mali DSM 20444 |
| CAGKPHEN_03081 | 2567319893 | transposase | 2597960139 | Bifidobacterium mongoliense DSM 21395 |
| CAGKPHEN_01539 | 2587086457 | **amino acid/polyamine/organocation transporter, APC superfamily** | 2594749849 | Staphylococcus aureus 12-03481 |
| CAGKPHEN_00701 | 2510425743 | 2,4-dienoyl-CoA reductase-like NADH-dependent reductase (Old Yellow Enzyme family) | 650074020 | Coprobacillus cateniformis 29_1 |
| CAGKPHEN_01811 | 2967887314 | 5-methylcytosine-specific restriction protein A | 2633520378 | Paenibacillus sp. FSL H7-0357 |
| CAGKPHEN_01788 | 2546478506 | Adenine-specific methyltransferase EcoRI | 2543464212 | Gardnerella vaginalis JCP7719 |
| CAGKPHEN_02517 | 8004821531 | diadenosine tetraphosphate (Ap4A) HIT family hydrolase | 2760253340 | Lactobacillus bombicola ESL0230 |
| CAGKPHEN_02990 | 2567307966 | EpsG family protein | 2514675952 | Desulfosporosinus sp. OT |
| CAGKPHEN_01771 | 2956994435 | hypothetical protein | 638426578 | Listeria monocytogenes 4b H7858 |
| CAGKPHEN_00388 | 2541042126 | hypothetical protein | 2515134932 | Amycolatopsis nigrescens CSC17Ta-90, DSM 44992 |
| CAGKPHEN_01375 | 2567318899 | methyltransferase, FkbM family | 2596378392 | Butyrivibrio sp. Su6 |
| CAGKPHEN_02821 | 2956993853 | hypothetical protein | 639864761 | Stigmatella aurantiaca DW4/3-1 |
| CAGKPHEN_02073 | 2510426317 | hypothetical protein | 638942375 | Vibrio sp. MED222 |
| CAGKPHEN_01752 | 2961475009 | hypothetical protein | 638426578 | Listeria monocytogenes 4b H7858 |
| CAGKPHEN_02818 | 2956996302 | hypothetical protein | 647774286 | Paenibacillus sp. D14 |
| CAGKPHEN_01359 | 8002251401 | IS30 family transposase | 2928611129 | Lactiplantibacillus pentosus 14.2.16 |
| CAGKPHEN_02680 | 2559135748 | NADPH:quinone reductase | 2635380075 | Rhodococcus koreensis DSM 44498 |
| CAGKPHEN_00233 | 2510421871 | **phospholipase A2-like protein** | 2504128148 | Peptoniphilus asaccharolyticus UW 228, DSM 20463 |
| CAGKPHEN_02898 | 2546483538 | Predicted ATP-dependent endonuclease of the OLD family, contains P-loop ATPase and TOPRIM domains | 2519759059 | Bacillus cereus MSX-A12 |
| CAGKPHEN_02061 | 645013308 | predicted ORF | 646247539 | Citrobacter portucalensis 30_2 |
| CAGKPHEN_02096 | 2521103886 | Protein of unknown function (DUF1492) | 638294946 | Lactobacillus phage A2 |
| CAGKPHEN_02088 | 2562510326 | Protein of unknown function (DUF1642) | 638294937 | Lactobacillus phage A2 |
| CAGKPHEN_01295 | 2515663251 | UDP-galactose 4-epimerase | 2615987991 | Bacillus sp. OK634 |
| CAGKPHEN_02117 | 2510425169 | putative phage tail component, N-terminal domain-containing protein | 638317177 | Lactobacillus phage Lc-Nu |
| CAGKPHEN_03030 | 2510425315 | **Restriction endonuclease S subunits** | 641316242 | Nitrosopumilus maritimus SCM1 |
| CAGKPHEN_02848 | 2967861424 | oxaloacetate decarboxylase, beta subunit | 2621086037 | Clostridium sulfidigenes 113A |
| CAGKPHEN_00141 | 2968369934 | uncharacterized protein (AIM24 family) | 2889491426 | Helcococcus massiliensis Marseille-P4590 |
| CAGKPHEN_00405 | 2510420295 | zinc ribbon protein | 648133082 | Butyrivibrio proteoclasticus B316 |
|  |  |  |  |  |
